# Supplementary material for: The fitness cost of spurious phosphorylation
Source: EMBO J. 2024 Sep 10;43(20):4720–51. doi: 10.1038/s44318-024-00200-7 (PMC11480408; doi:10.1038/s44318-024-00200-7)
Supplement: Supplementary file 1 — Appendix [file 44318_2024_200_MOESM1_ESM.pdf]

The fitness cost of spurious phosphorylation

Appendix supplementary figures

Table of Contents

Appendix Figure S1	pg 2
Appendix Figure S2	pg 3
Appendix Figure S3	pg 4-5
Appendix Figure S4	pg 5-6
Appendix Figure S5	pg 6
Appendix Figure S6	pg 7
Appendix Figure S7	pg 8-9
Appendix Figure S8	pg 10
Appendix Figure S9	pg 11
Appendix Figure S10	pg 12
Appendix Figure S11	pg 13
Appendix Figure S12	pg 14-15
Appendix Figure S13	pg 16
Appendix Figure S14	pg 17
Appendix Figure S15	pg 18-19
Appendix Figure S16	pg 20-21
Appendix Figure S17	pg 22
Appendix Figure S18	pg 23
Appendix Figure S19	pg 24
Appendix Figure S20	pg 25
Appendix Figure S21	pg 26
Appendix Figure S22	pg 27
Appendix Figure S23	pg 28-29
Appendix Figure S24	pg 30
Appendix Figure S25	pg 31

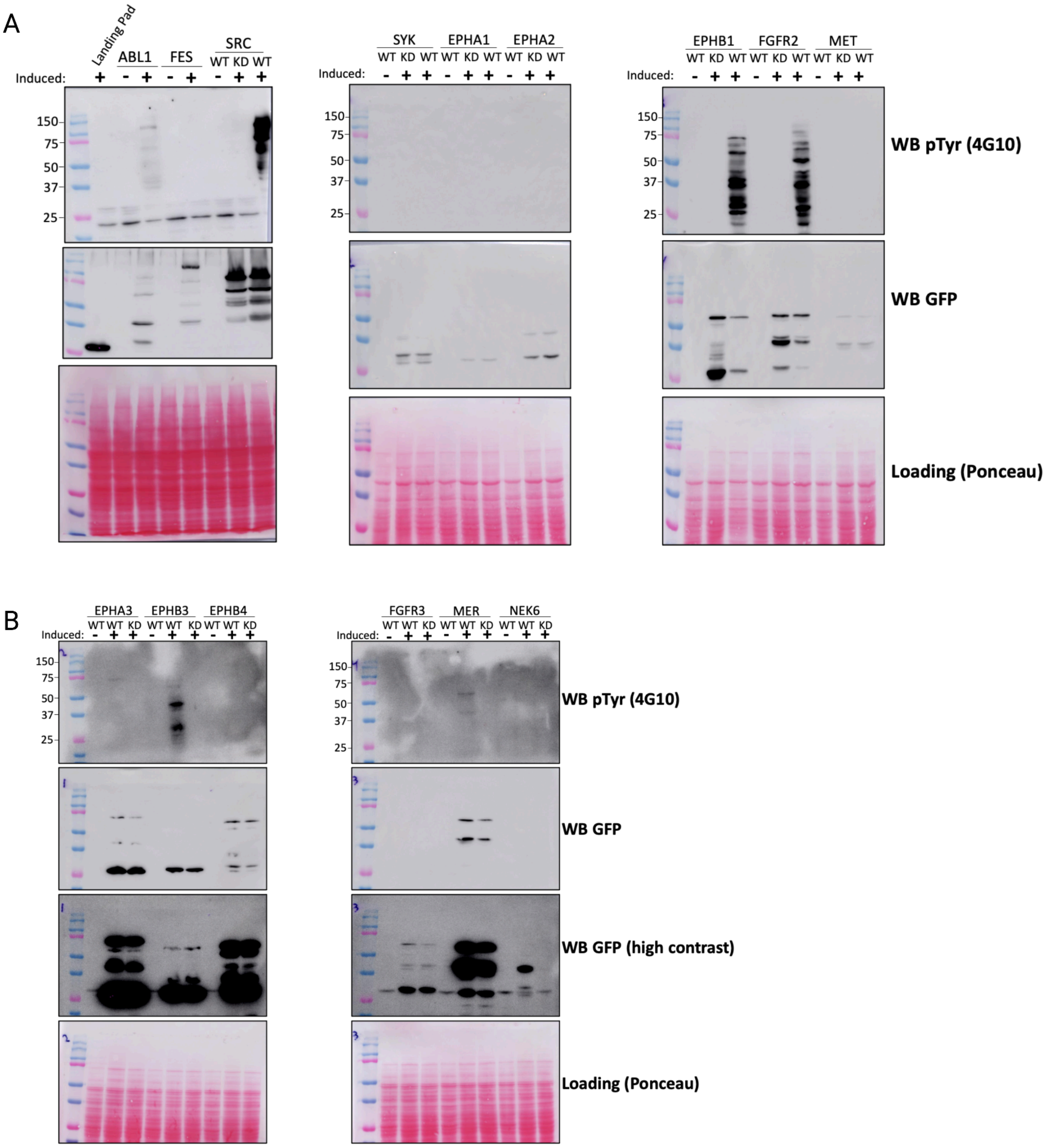

Appendix Figure S1: p-Tyr activity of expressed kinases in yeast. Yeast cells were grown with (+) or without (-) induction to check for p-Tyr activity. Western blots to detect p-Tyr (pTyr (4G10)) and GFP (kinase expression) were done. **A)** ABL1, FES, SRC, SYK, EPHA1, EPHA2, EPHB1, FGFR2 and MET. In the left-most panel, a small sample of kinases (ABL1, FES, and SRC) are compared to an empty landing pad (first lane) as a control. **B)** EPHA3, EPHB3, EPHB4, FGFR3, MER and NEK6. WT: wild-type kinase. KD: kinase-dead mutant. Figure was created with Biorender.com.

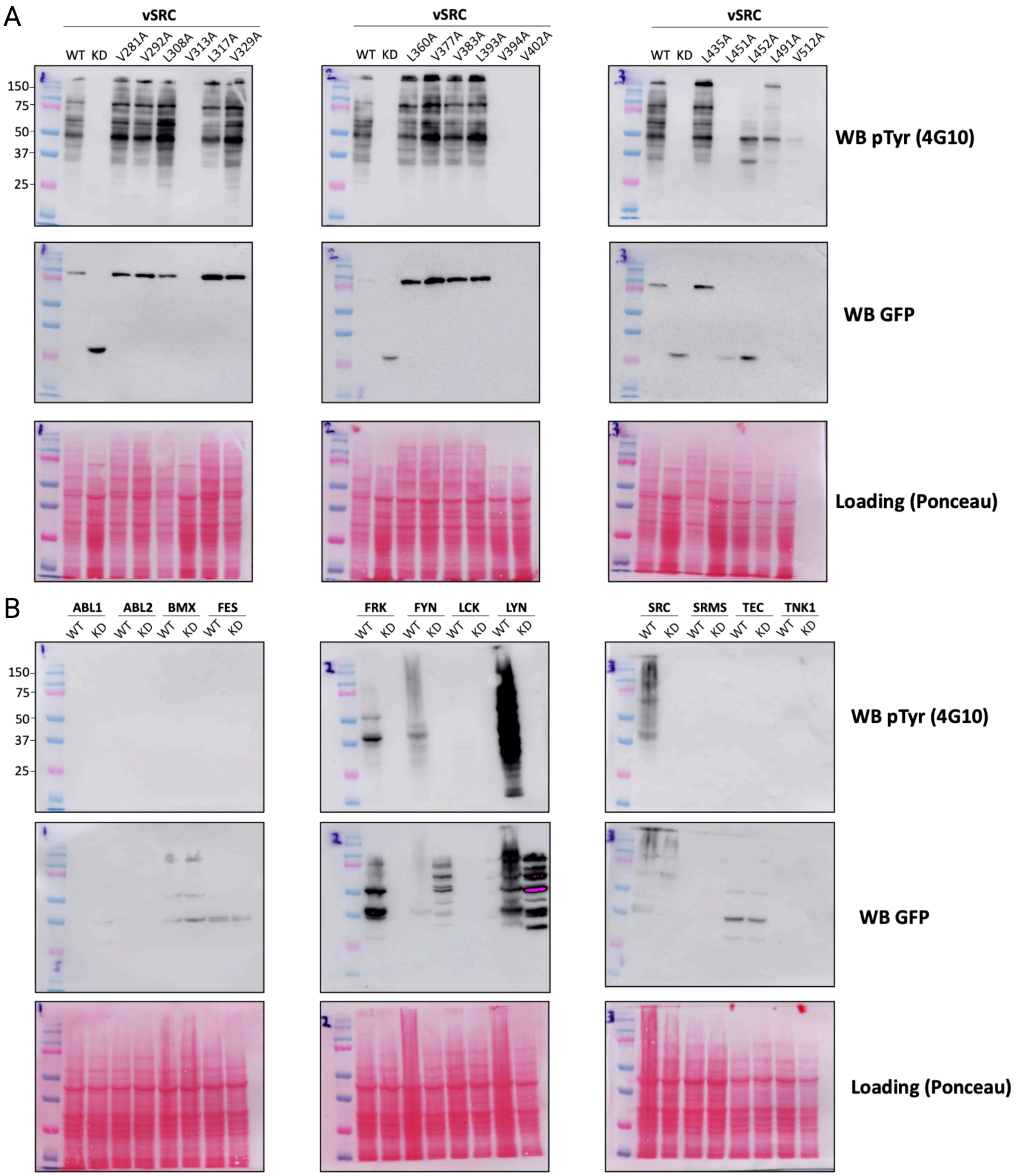

Appendix Figure S2: p-Tyr activity of expressed kinases in yeast. Yeast cells were grown with induction to check for p-Tyr activity. Western blots to detect p-Tyr (pTyr (4G10)) and GFP (kinase expression) were done. **A)** vSRC and its mutants **B)** ABL1, ABL2, BMX, FES, FRK, FYN, LCK, LYN, SRC, SRMS, TEC and TNK1. WT: wild-type kinase. KD: kinase-dead mutant. The vSRC mutants V313A, V402A, V512A, and V377A were later found to contain non-synonymous mutations in the coding sequence (via sequencing) and so were excluded from any further analysis. Figure was created with Biorender.com.

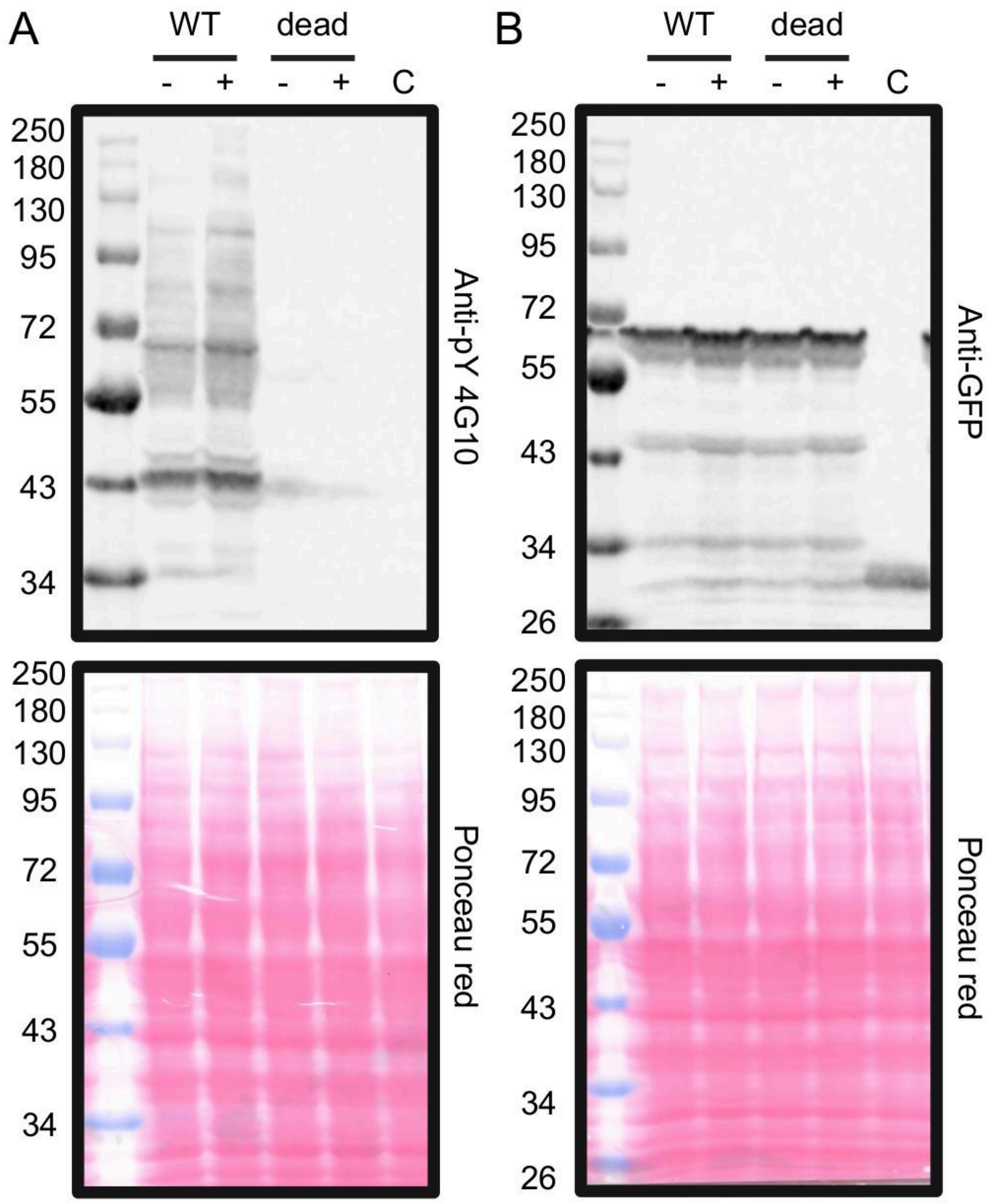

Appendix Figure S3: examining the effect of a phosphatase inhibitor on p-Tyr levels following the expression of a human tyrosine kinase. Effect of phosphatase inhibitor on phospho-tyrosine levels. EPHB1 WT or dead kinase were first grown for 5h, then kinase expression was induced for 2h with 25 nM estradiol. At the same time, the culture was split in two, one part was exposed to PhosStop (D-Mannitol, Sodium molybdate dihydrate, Sodium orthovanadate, Cantharidine) (+) (Sigma, 4906845001) and the second part was not (-). Protein extracts were separated on a 10% acrylamide gel and transferred to a nitrocellulose membrane. Proper loading and transfer was confirmed with Ponceau red. Tyrosine phosphorylation (A) was detected with the anti-4G10 from Sigma (05-321). Kinase expression (B) was detected with an anti-GFP

(Roche, 11814460001). Empty landing pad was used as a negative control (c). Figure was created with Biorender.com.

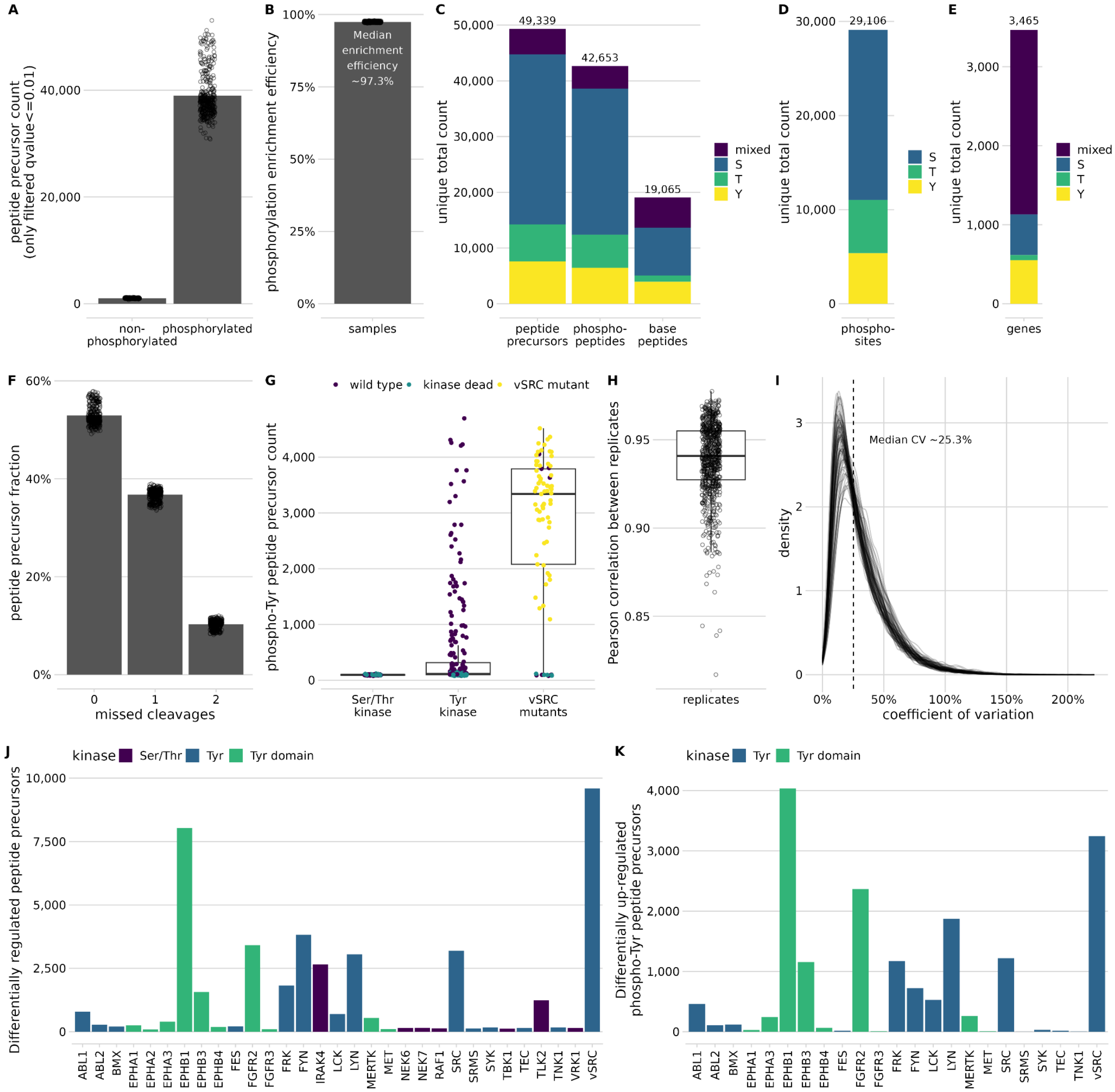

Appendix Figure S4: Phosphoproteomics quality control. 386 phospho-enriched yeast samples of 78 conditions (five replicates each, see methods section) were measured by mass spectrometry and processed by FragPipe/DIA-NN. **A-B)** The vast majority of identified peptide precursors are phosphorylated, yielding a median enrichment efficiency of 97.3%. For these two plots, the data was only filtered using local q-value ≤ 0.01. **C-E)** Total unique identifications on peptide precursor (PEPT(ph)IDEK_2), phosphopeptide (PEPT(ph)IDEK), base peptide (PEPTIDEK), phospho-site (Ser(ph)-123 on gene 1) and gene level. **F)** Digestion efficiency as shown by the number of missed cleavages per peptide precursor. **G)** Phosphotyrosine containing peptide precursors are identified almost exclusively in tyrosine kinase wild type and vSRC wild type / mutant conditions. **H)** Pearson correlation is shown between

biological replicates of the same conditions. **I)** Density graph showing the coefficients of variation for the 5 biological replicates per condition. **J-K)** SAM statistical significance testing shows **J)** the up- and down-regulated peptide precursors for all serine/threonine and tyrosine kinases, as well as **K)** the up-regulated peptide precursors containing phospho-tyrosine for the tyrosine kinases.

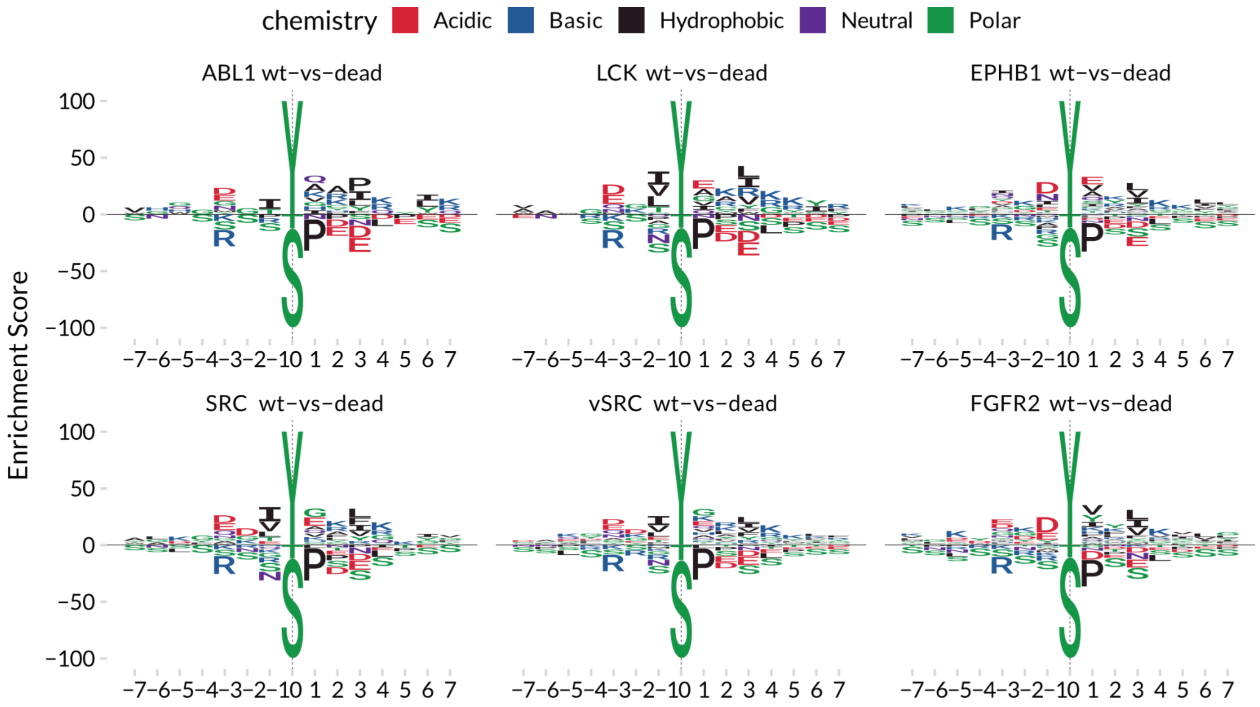

Appendix Figure S5: Kinase substrate motif enrichment analysis of selected phospho-tyrosine kinases. To test for kinase activity, kinase substrate motif enrichment analysis was performed on phospho-tyrosine containing peptides vs all other phospho-peptides using the R package dagLogo (Ou *et al*, 2020). By using only phosphorylated tyrosine peptides as the foreground, it could be assured that they must have been phosphorylated by the respective kinase. Amino acid positions were filtered for significance using a p-value threshold ≤ 0.05. The generated kinase substrate motifs recapitulate known kinase substrate preferences as for ABL1 (Colicelli, 2010), LCK and SRC (Shah *et al*, 2018).

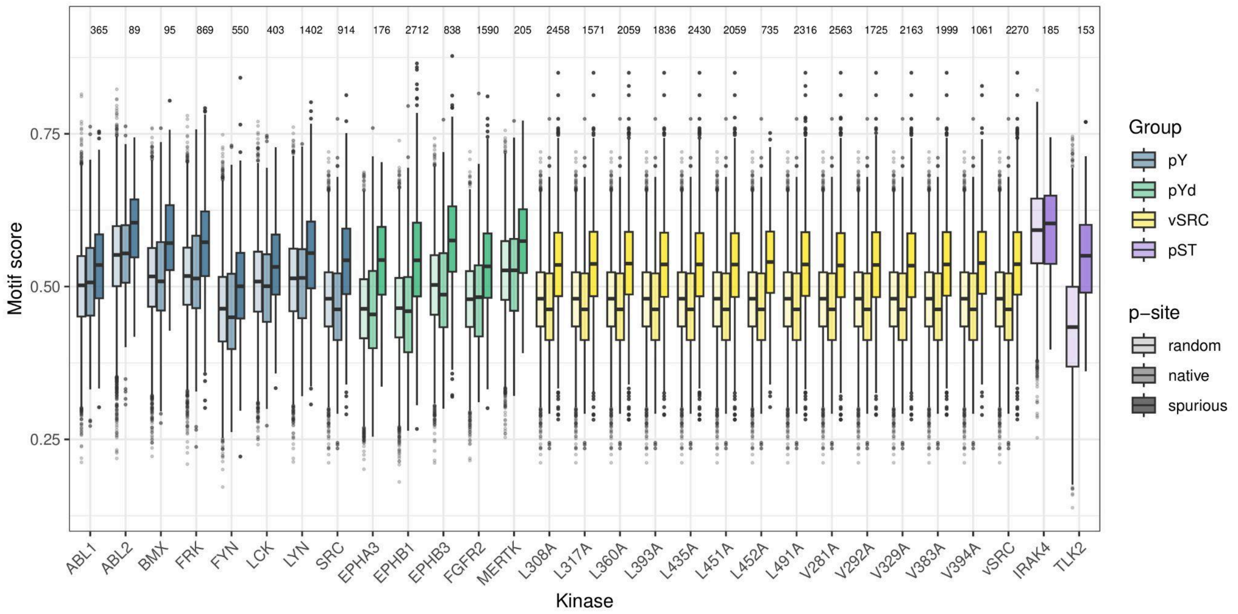

Appendix Figure S6: Spuriously phosphorylated sites have a better match to the cognate kinase motif than expected by chance. The flanking region (positions -5 to +5) of each spurious phosphorylation site was scored against a position weight matrix (PWM) of its cognate kinase. The normalised motif score (y-axis) has a maximum value of 1 (perfect match to the kinase PWM) and minimum value of 0 (worst possible match to the kinase PWM). For each kinase, this analysis was repeated on n=169 endogenous pY sites and n=10,000 random pY sequences (positions -5 to +5). The S/T kinases IRAK4 and TLK2 were scored only against their cognate spurious pS/pT sites and n=10,000 random pS/pT sequences (positions -5 to +5). Tyrosine kinase PWMs were derived from (Sugiyama *et al*, 2019), and the IRAK4 and TLK2 PWMs were sourced directly from (Johnson *et al*, 2023).

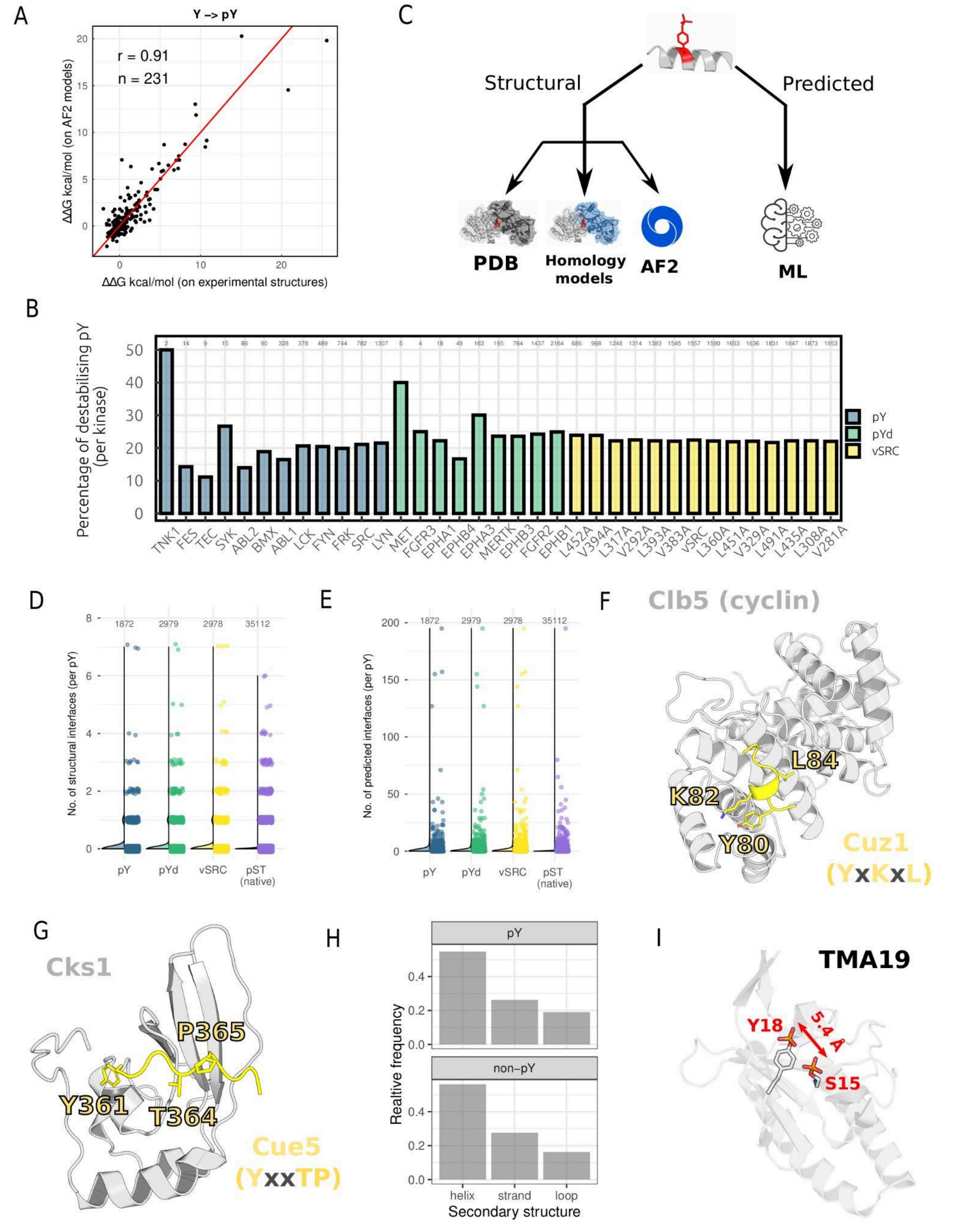

Appendix Figure S7: Structural overview of spurious phosphorylation across the proteome. A) Correlation of the FoldX ΔΔG kcal/mol (Y to pY) predictions between

PDB structures and their corresponding AF2 models for spurious pY substrates. A resolution cut-off of 3Å was applied to the PDB models. **B)** For all spurious pY per kinase, the percentage predicted to be destabilising for the substrate using a ΔΔG threshold of 2 kcal/mol **C)** Spurious pY sites were mapped to structural interfaces corresponding to PDB structures (wwPDB consortium, 2019), homology models (Mosca *et al*, 2013), and AF2-based models of protein interactions (Humphreys *et al*, 2021). Interfaces of protein-protein interactions were also predicted via machine learning (Meyer *et al*, 2018). Panel created with the help of BioRender.com. **D)** The number of unique interfaces (per pY) found in structural models (PDB, homology, or AF2). **E)** The number of unique interfaces (per pY) predicted via machine learning (interactomeINSIDER) (Meyer *et al*, 2018). **F)** AlphaFold multimer-based model of Clb5 cyclin binding to a peptide containing Y80 and the R/K-x-L cyclin docking motif. **G)** AlphaFold multimer-based model of Cks1 binding to a Y361 and the Cks1 docking motif (T-P). **H)** Secondary structure profile predicted by DSSP (Kabsch & Sander, 1983)**,** between spurious pY sites and non-phosphorylated sites on the same protein (non-pY). **I)** 3D distance between the native phosphosite pS15 and the spurious phosphosite pY18 in the AF2 model of TMA19.

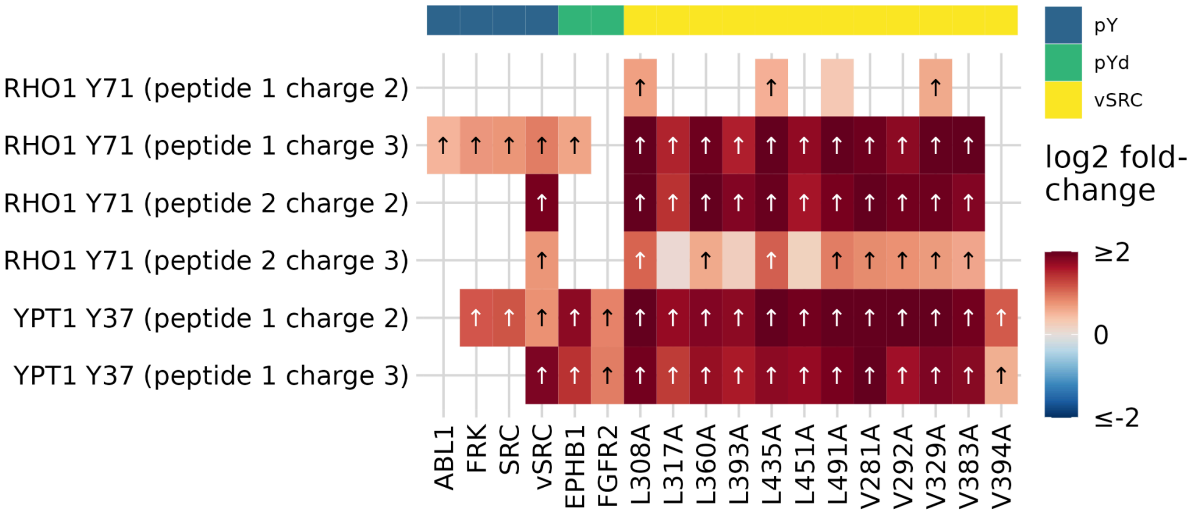

Appendix Figure S8: Spurious phosphorylation of sites homologous to pY32 and pY64 in KRAS. Phosphorylation of Y32 and Y64 in KRAS has an inhibitory effect on Ras signalling (Kano *et al*, 2019; Wang *et al*, 2021). Rho1 pY71 and Ypt1 pY37 are homologous phosphosites in *S. cerevisiae.* The log2-fold change is indicated by the colour bar, and the up-arrow indicates significant upregulation (WT-dead). pY: full-length tyrosine kinase, pYd: tyrosine kinase domain, vSRC: WT VSRC and its mutants.

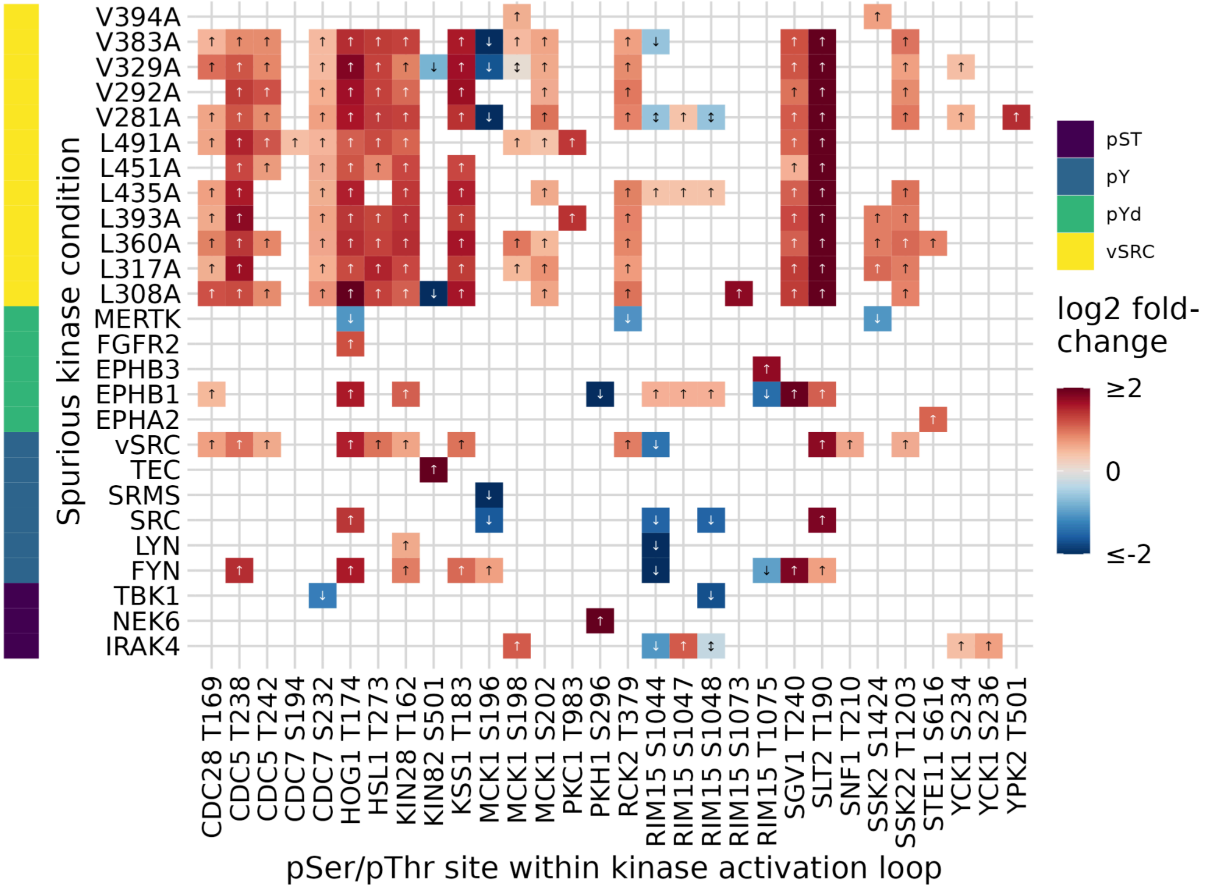

Appendix Figure S9: S/T phosphorylation on yeast kinase activation loops. Down- and up-regulation (WT-dead) of pS/pT sites mapping to kinase activation loops. Data is arranged in terms of the human spurious kinase (rows) and the native yeast kinases (columns) that the phosphosites map to. The log2 fold-change (WT-dead) is indicated by the colour bar, and significant up- and down-regulation is indicated by ‘up’ and ‘down’ arrows. pY: full-length tyrosine kinase, pYd: tyrosine kinase domain, vSRC: WT vSRC and its mutants.

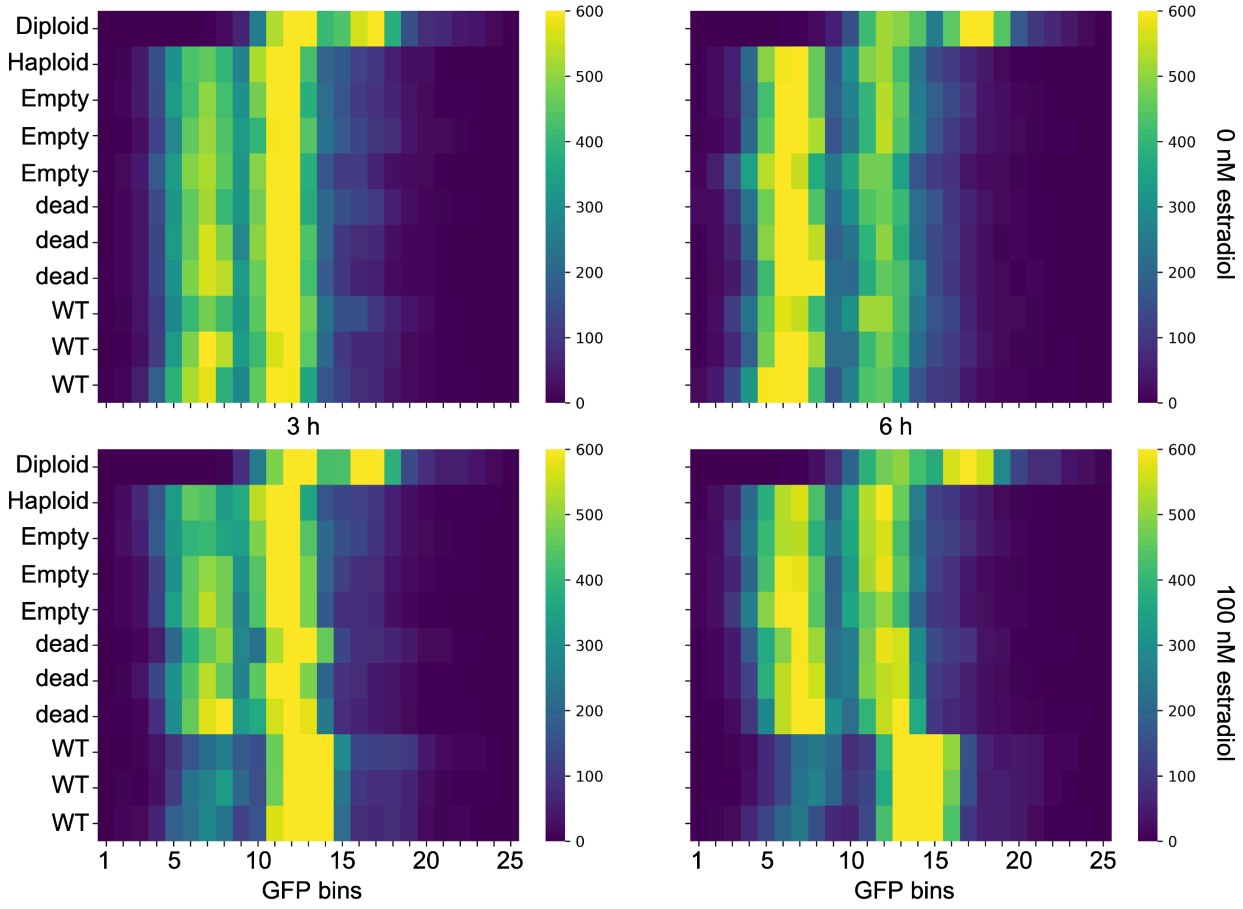

Appendix Figure S10: Effect of vSRC expression on ploidy level. vSRC WT or dead were expressed (100 nM estradiol) or not (0 nM estradiol) for 3 or 6 hours. Empty landing pad, haploid and diploid strains were grown in the same conditions. All strains were grown in three independent replicates. Cells were fixed in ethanol 70%. RNA was removed from the cells with an overnight incubation with RNAse A. Finally, DNA content was stained overnight with Sytox Green. After staining, DNA content was registered for 5,000 cells in a Guava EasyCyte HT (Guava EasyCyte instrument, Cytek Bio). Figure was created with Biorender.com.

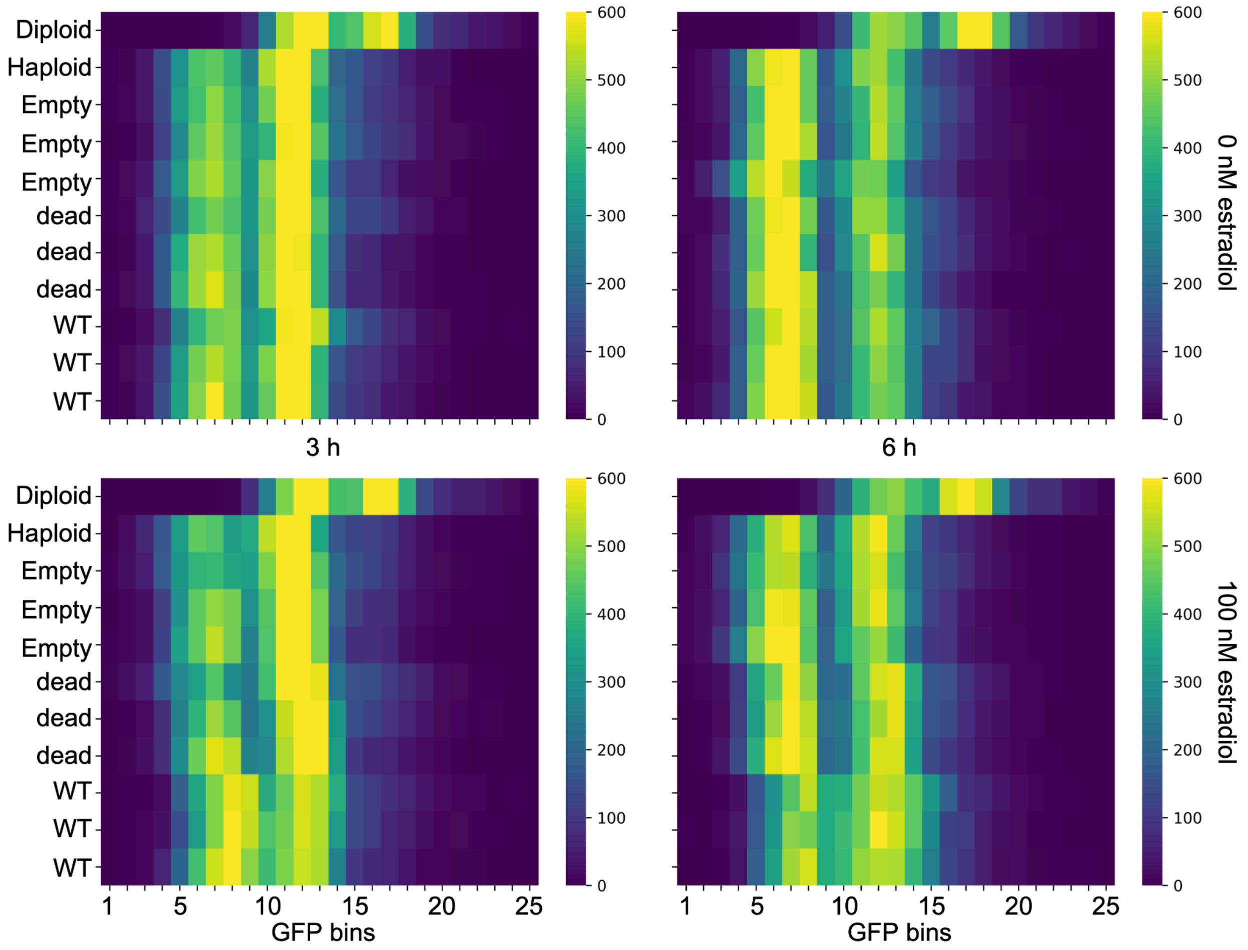

Appendix Figure S11: Effect of EPHB1 expression on ploidy level. EPHB1 WT or dead were expressed (100 nM estradiol) or not (0 nM estradiol) for 3 or 6 hours. Empty landing pad, haploid and diploid strains were grown in the same conditions. All strains were grown in three independent replicates. Cells were fixed in ethanol 70%. RNA was removed from the cells with an overnight incubation with RNAse A. Finally, DNA content was stained overnight with Sytox Green. After staining, DNA content was registered for 5,000 cells in a Guava EasyCyte HT (Guava EasyCyte instrument, Cytek Bio). Figure was created with Biorender.com.

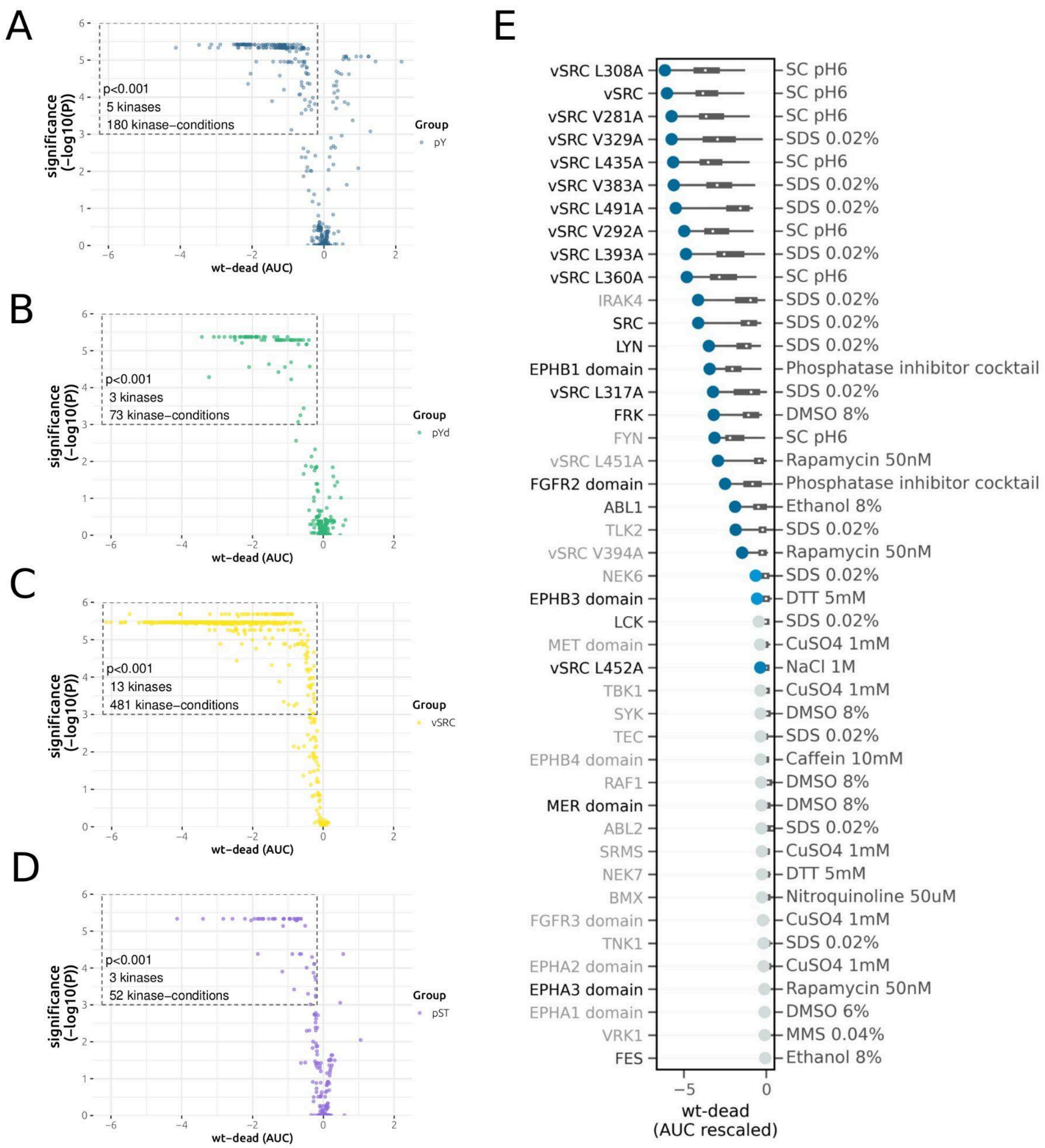

Appendix Figure S12: The effect of kinase expression (WT-dead) on fitness. Volcano plots for the fitness effect of different kinases separated by group: **A)** full-length tyrosine kinases (pY), **B)** tyrosine kinase domains (pYd), **C)** v-SRC and its mutants (vSRC), and **D)** human serine/threonine kinases (pST). The x-axis represents the difference in area under the curve (AUC) between growth curves for the WT kinase and corresponding kinase-dead mutant. The y-axis represents the FDR-adjusted p-value for the significance of the AUC difference between the WT and dead kinase. All kinase-condition pairs are represented in each volcano plot. **E)** The fitness scores

(x-axis) of all the kinases (y-axis) measured from the fitness screen are shown. The condition with maximum deleteriousness, marked by a circular dot is indicated on the right side of the plot. The colours of the dots indicate the statistical significance of the fitness score: dark blue indicates $P<0.001$, light blue indicates $0.001<P<0.05$ and grey indicates non-significance. The distribution of the fitness scores in all conditions is shown by the horizontal grey box plots. The colours of the kinases indicate their activity level from Western blotting analysis **(Appendix Figures S1 and S2)**: shown in black are kinases that were found to be active, grey indicates weakly active, and light grey indicates kinases that showed no activity.

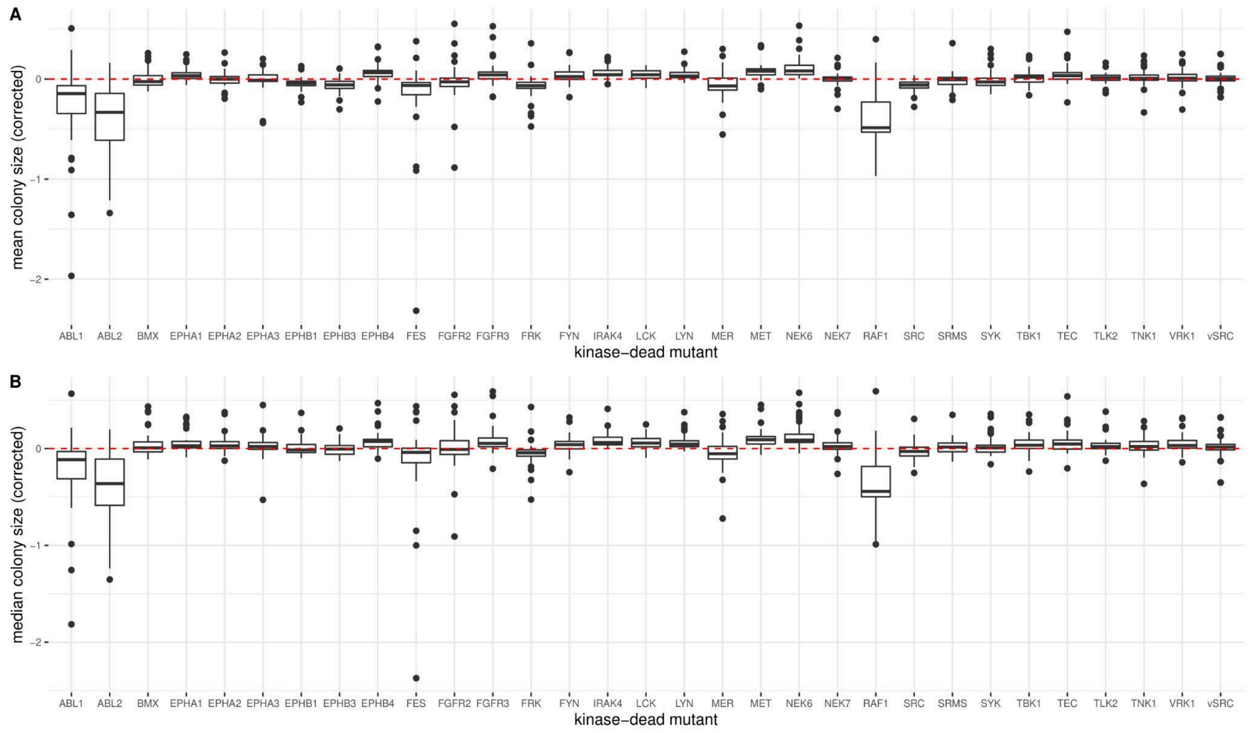

Appendix Figure S13 The effect of expression of the kinase-dead mutant on growth (colony size). The mean (top) and median (bottom) colony size for the kinase-dead mutant strains. Each colony size has been scaled with respect to a reference strain not expressing the kinase coding sequence (see Methods).

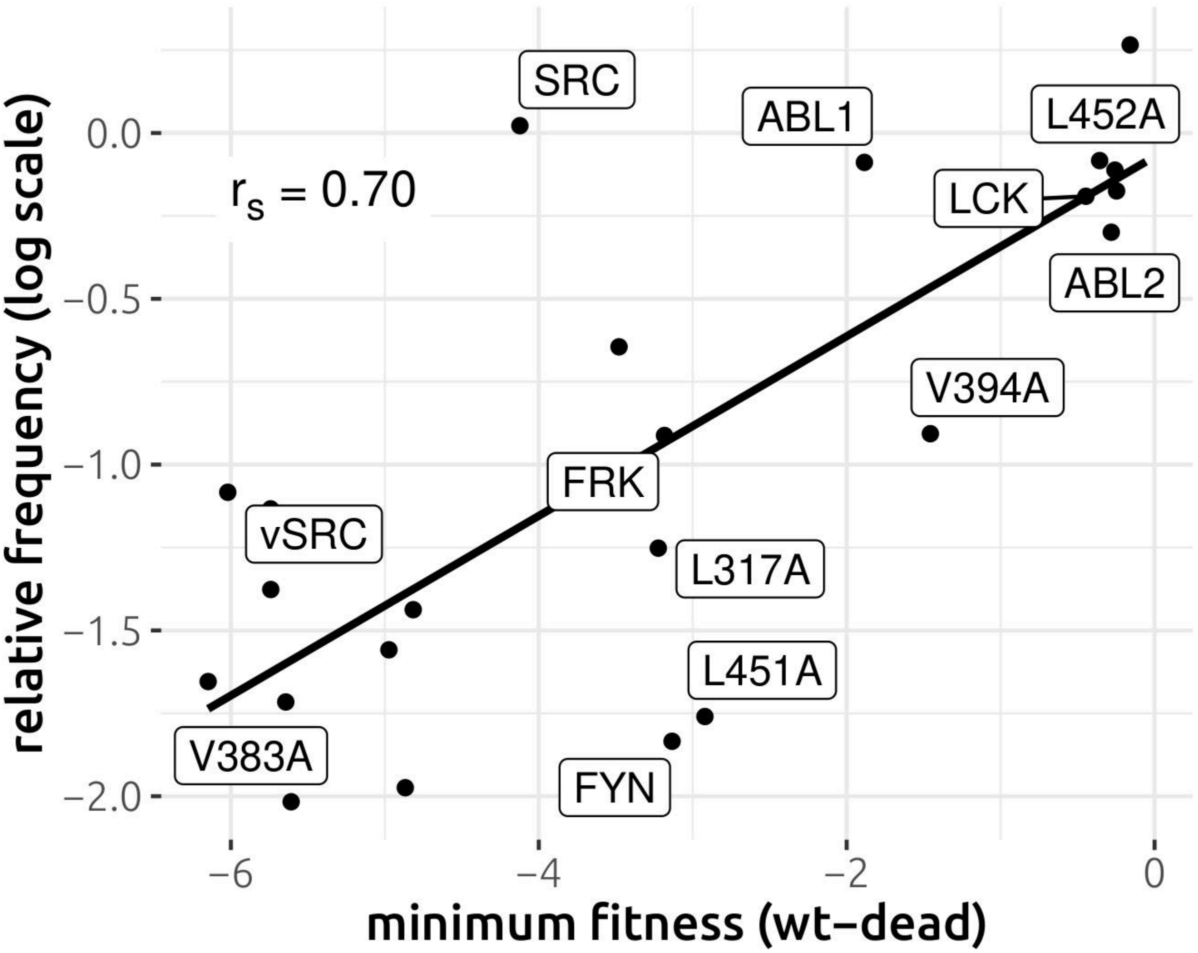

Appendix Figure S14: Correlation of results from direct competition assays (y-axis) with minimum fitness determined from colony sixes (x-axis). Correlation between WT-dead fitness inferred from colony sizes (x-axis), and relative growth between the kinase and empty landing-pad control (y-axis). The relative frequencies were log-scaled and taken at the final time point (72 hours) of the competition assay (see Methods).

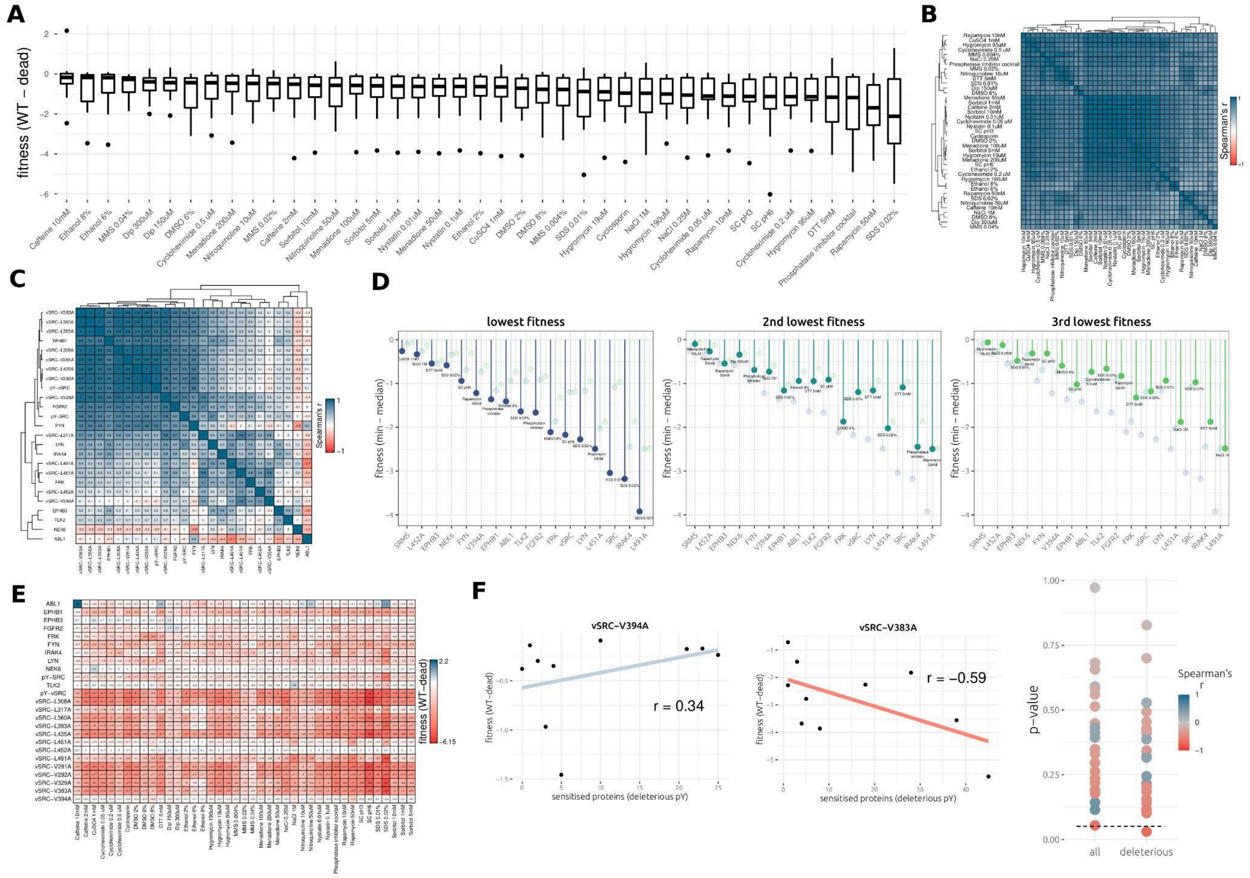

Appendix Figure S15: The difference in fitness effects between conditions. A) The fitness effects per condition across different kinases. To minimise redundancy, data for non-redundant vSRC mutants (WT, V491A, L451A, L452A, and V394A) and only one inactive kinase (SRMS) were used to generate the boxplots, alongside all active kinases. **B)** Correlation of fitness profiles (across kinases) between different conditions. Only kinases with at least one significant fitness defect were used to generate the correlation matrix. **C)** Correlation of fitness profiles (across conditions) between different kinases. Only kinases with at least one significant fitness defect were used to generate the correlation matrix. **D)** For each non-redundant kinase in the dataset, the difference between the minimum fitness and the median fitness across conditions. The second and third lowest fitnesses are represented in the middle and right panels, respectively. **E)** Heatmap of fitness scores (WT-dead) across all active kinases (rows) and tested conditions (columns). **F)** Cross-examination between kinase phosphosites detected in this study and the conditionally sensitive KO genes identified in (Viéitez *et al*, 2022). For the 10 conditions that overlap between this study and (Viéitez *et al*, 2022), the WT-dead fitness (y-axis) is taken and compared with the number of (predicted) deleterious pY mapping to conditionally sensitive proteins for each condition. (left) for the kinase vSRC V394A, with each point representing one condition. (middle) for the kinase vSRC V383A, with each point representing one condition. (right) The p-value and correlation coefficient for each regression, with each point representing one kinase. This is given for a correlation across ‘all’ pY that map to conditionally sensitive genes and for predicted ‘deleterious’ pY only. Significant negative correlations are expected if the

differential fitness effects can be explained by loss-of-function (LOF) pY mapping to conditionally sensitive proteins.

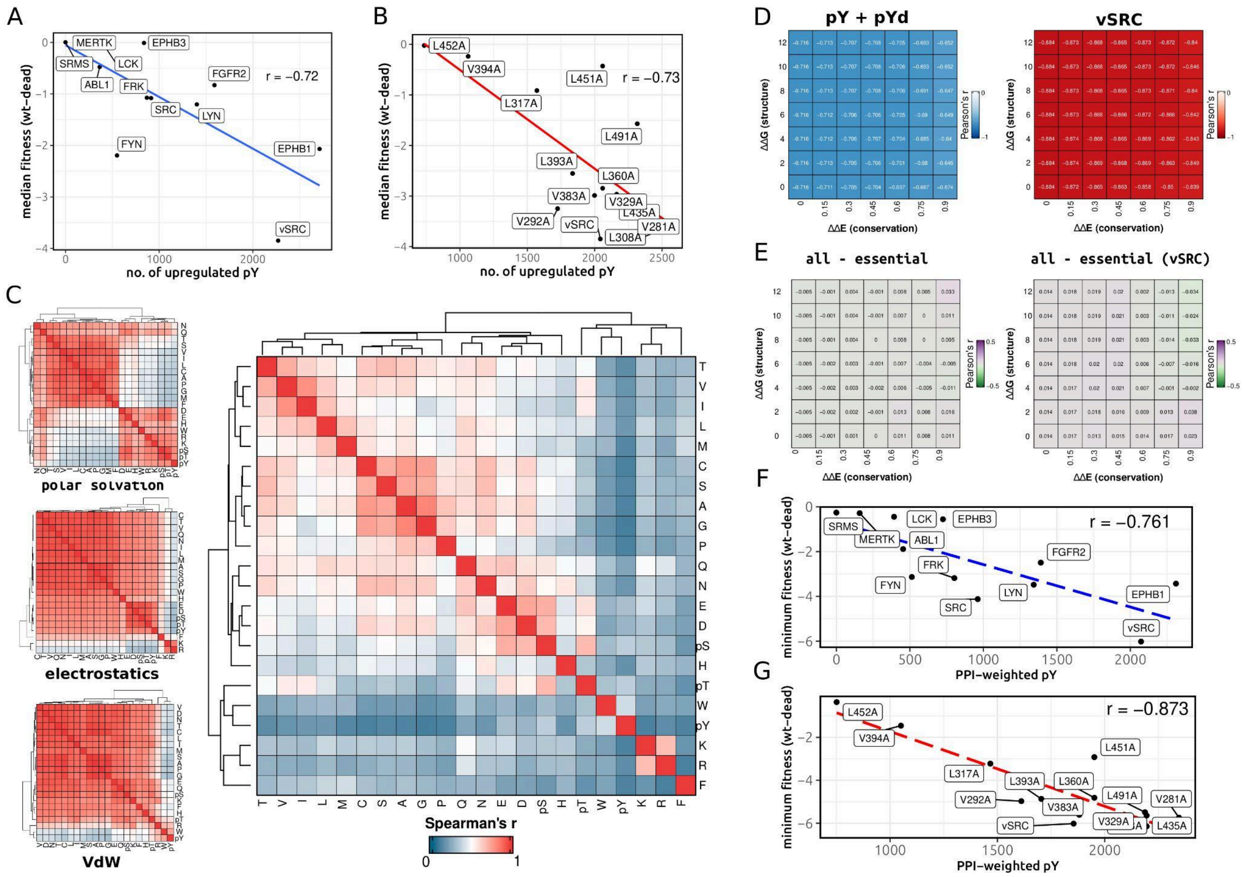

Appendix Figure S16: Relationship between kinase toxicity and phosphosite properties. A) Correlation between median fitness (across conditions, WT-dead) and the number of significantly upregulated pY sites (WT-dead) for each non-redundant kinase in the dataset. **B)** Correlation between median fitness (across conditions, WT-dead) and the number of significantly upregulated pY sites (WT-dead) for WT vSRC and the mutants generated in this study. **C)** For a large sample of spurious pY sites (n = ~2300**)**, the modified Y residue was mutated *in silico* to pY, pS, pT and the 19 other amino acids. Energy terms for the modification (Y to mutant/phosphosite) were calculated across all tested pY sites including polar solvation (top), electrostatic (middle), and Van der Waals (lower) energy terms. 22x22 correlation matrices were generated for each energy term to measure the similarity in effects of mutation and phosphorylation across various biophysical parameters. The mean correlation coefficient between energy terms was then taken to give an estimate of the average similarity between the 19 amino acids and pS, pT, and pY (see *Methods*). **D)** Correlation between minimum fitness (across conditions, WT-dead) and the number of significantly upregulated pY sites (WT-dead) for each non-redundant kinase in the dataset. Each value in the grid gives the Spearman's correlation coefficient after filtering for pY sites on the basis of their destabilising effect (ΔΔG) or on the conservation of the modified residue (ΔΔE). Left: for non-redundant pY and pYd kinases. Right: for vSRC and its mutants. **E)** The same protocol is applied as in panel **D,** but this time the heatmap gives the *difference* in correlation coefficient using all spurious pY substrates against using essential proteins only (all - essential). **F)** Correlation between minimum fitness (across

conditions, WT-dead) and the number of significantly upregulated pY sites (WT-dead) for each non-redundant kinase in the dataset. Each spurious pY is weighted so that pY predicted to map to many interfaces have a higher weight than non-interacting pY or those mapping to a small number of interfaces. **G)** The same correlation as in panel *F* but for vSRC and its mutants.

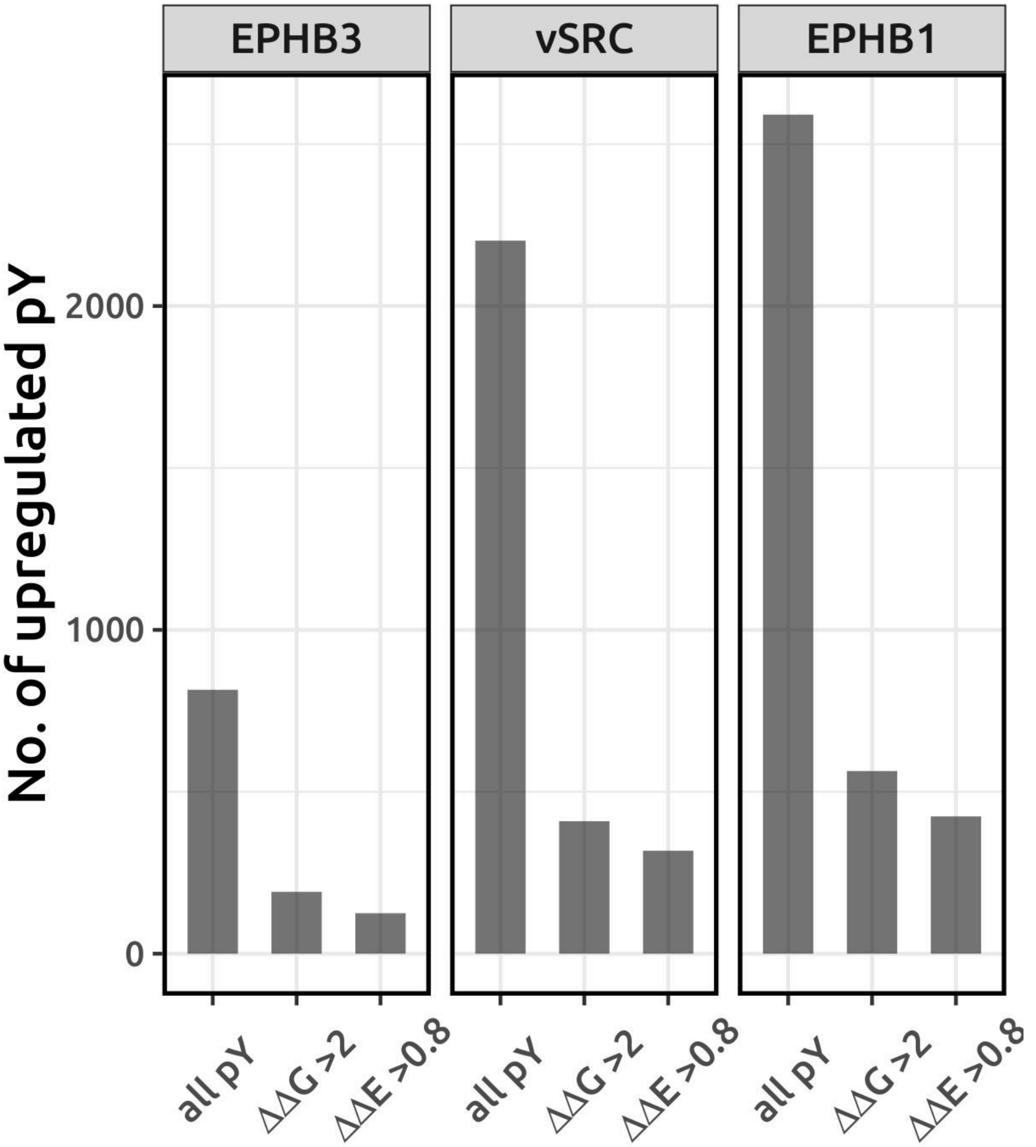

Appendix Figure S17: Spurious phosphorylation annotation for EPHB3, vSRC, and EPHB1. For EPHB3, vSRC, and EPHB1, the total number of upregulated pY, the number of destabilising pY (protein level, $\Delta\Delta G > 2$)**,** and the number of pY mapping to highly conserved positions ($\Delta\Delta E > 0.8$). EPHB3 is an active kinase but with only a small effect on fitness **(Figure 4D, Appendix Figure S16A)**. vSRC and EPHB1 are among the kinases most deleterious for fitness, have the strongest pY profiles, and are used here to infer phosphorylation stoichiometry **(Figure 4D, Figure 4G, Figure EV4, see Methods).**

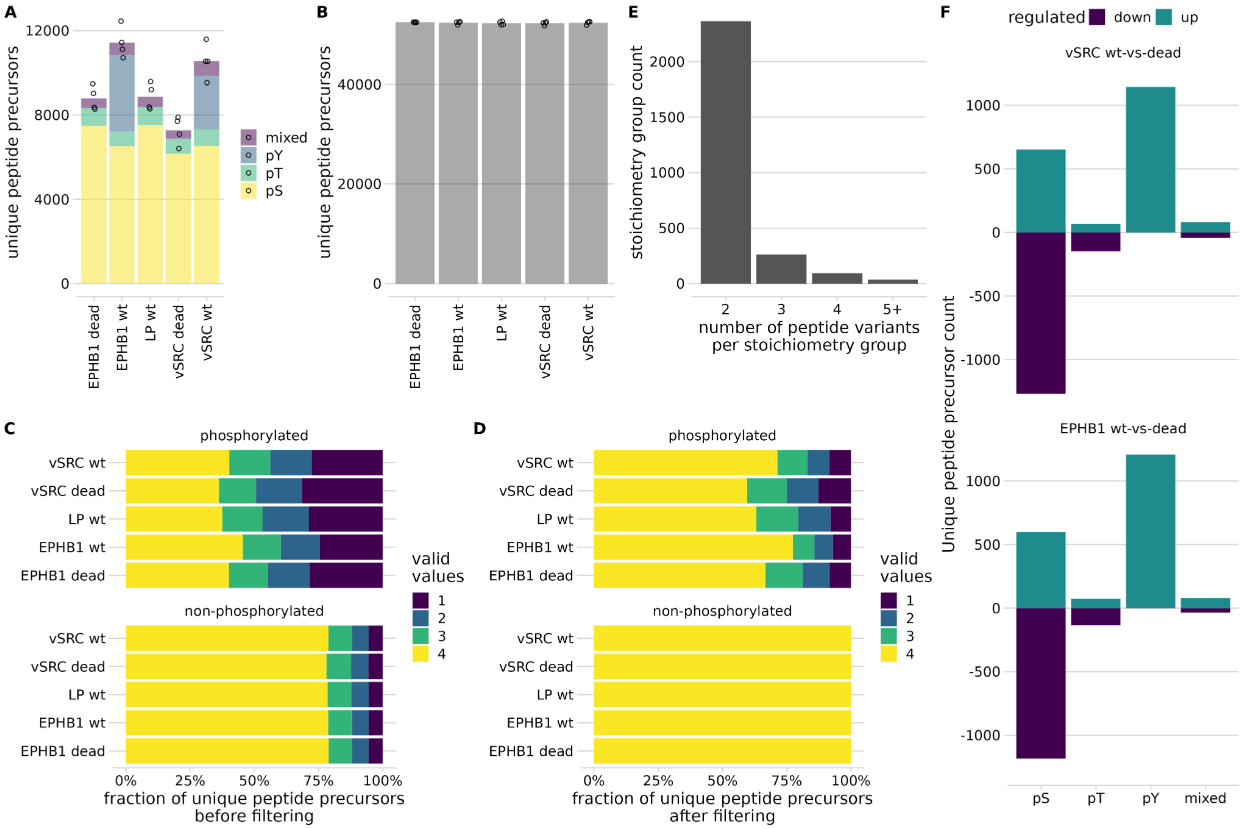

Appendix Figure S18: Calculation of stoichiometry values from (phospho-)proteomics data. A-V) Unique peptide precursor count of **A)** phosphoproteomic and **B)** proteomic data. **C-D)** The fraction of peptide precursors that have been quantified in 1 to 4 replicates (= valid values) per condition **C)** before and **D)** after quality control filtering. **E)** For stoichiometry groups with three or more peptide precursor variants, two or more isoforms of phosphorylated peptides were quantified. To simplify the correlation modelling, these stoichiometry groups were discarded. **F)** LIMMA analysis of phosphorylated peptide precursors reveals the same regulatory pattern of highly up-regulated phosphotyrosine containing peptide precursors, the non-phosphorylated counterparts of which were discarded for protein-level normalisation.

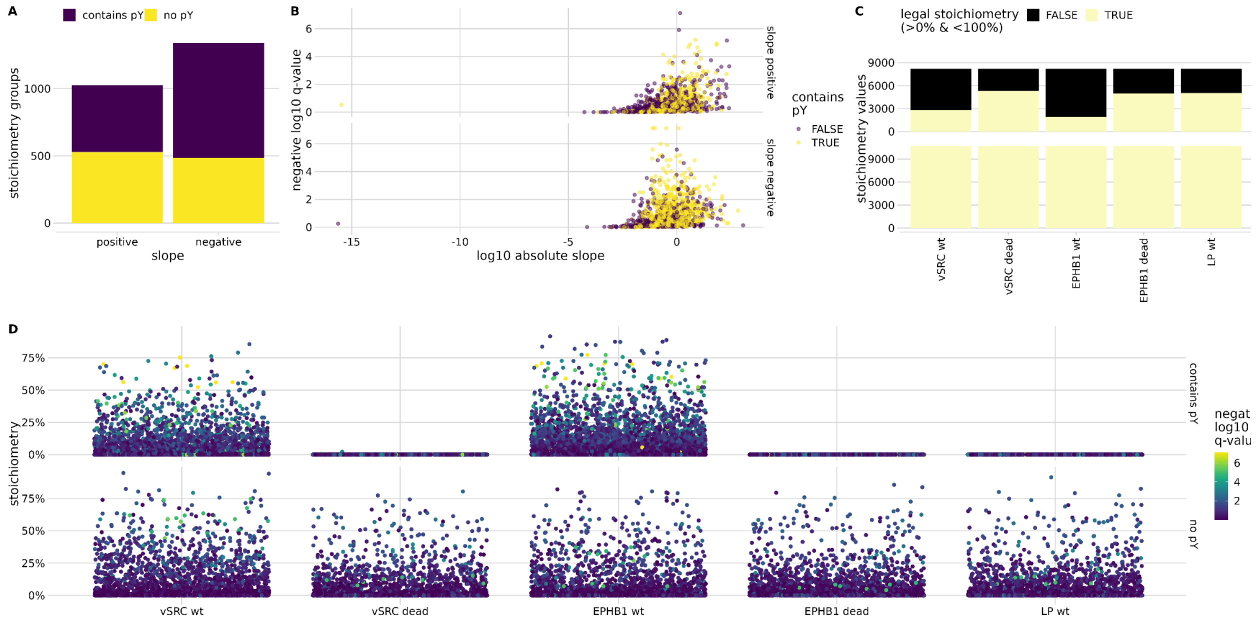

Appendix figure S19 Quality control and filtering of stoichiometry values. A) A negative slope during stoichiometry group correlation is required for unambiguous stoichiometry value estimation. Peptide precursors containing phosphotyrosine are enriched in this fraction, most likely because they show higher absolute regulation between conditions. **B)** In the same manner, phosphotyrosine containing stoichiometry groups with negative slopes yield higher negative log 10 q-values. **C)** Stoichiometry groups with positive slopes can yield stoichiometry values below 0% or above 100% (termed “illegal”), and are therefore entirely discarded. **D)** The vast majority of peptide precursors containing phospho-tyrosine with significantly up-regulated stoichiometry values can be found in the kinase wild type conditions, as expected.

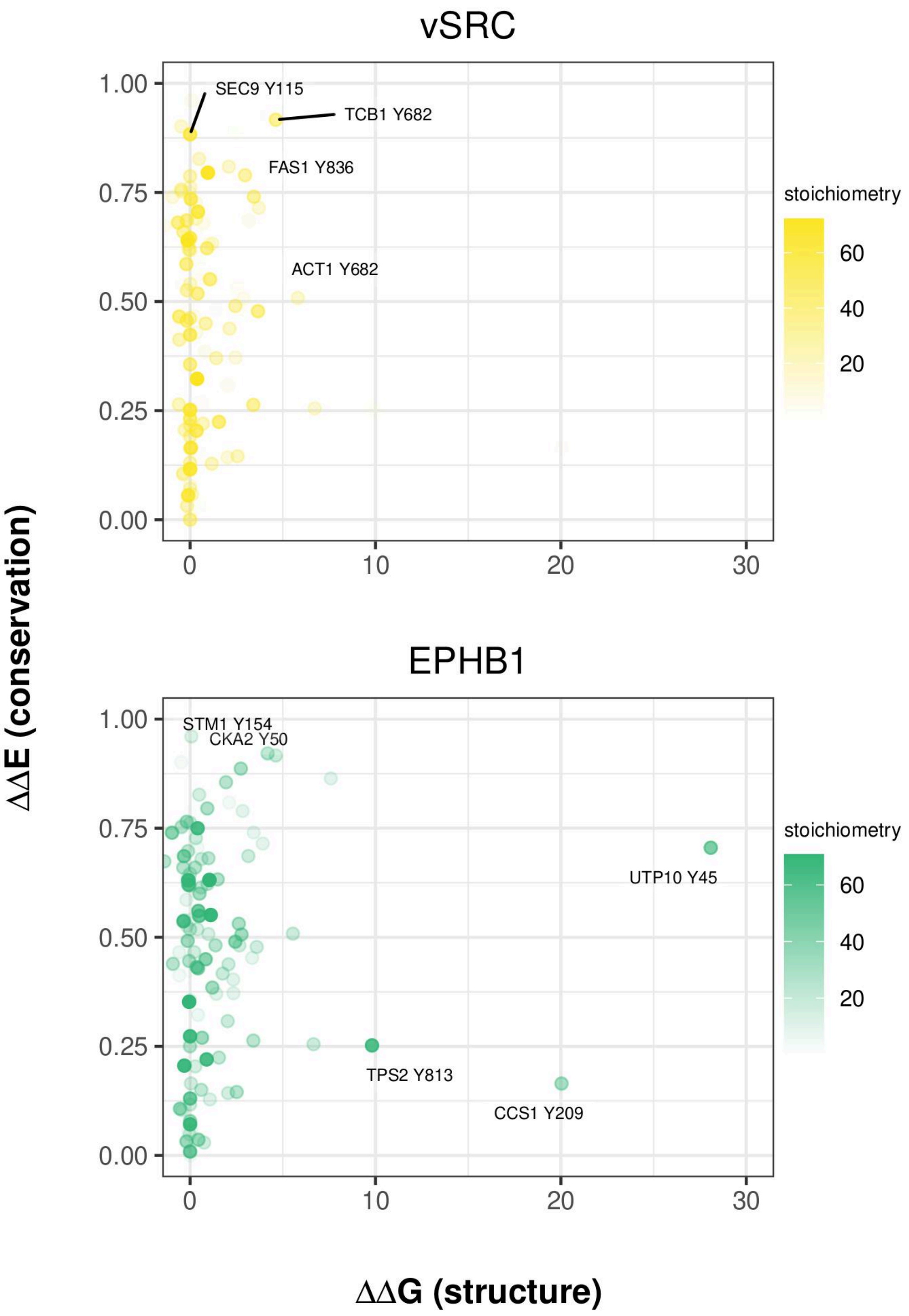

Appendix Figure S20: Stoichiometry of vSRC and EPHB1 pY sites that are predicted to be deleterious for function. Higher ΔΔG values correspond to more destabilising pY (protein-level) whereas higher ΔΔE values correspond to pY mapping to more conserved Y positions. Higher inferred stoichiometries are given in dark yellow/green and lower inferred stoichiometries are given in light yellow/green. Top: vSRC pY sites. Bottom: EPHB1 pY sites.

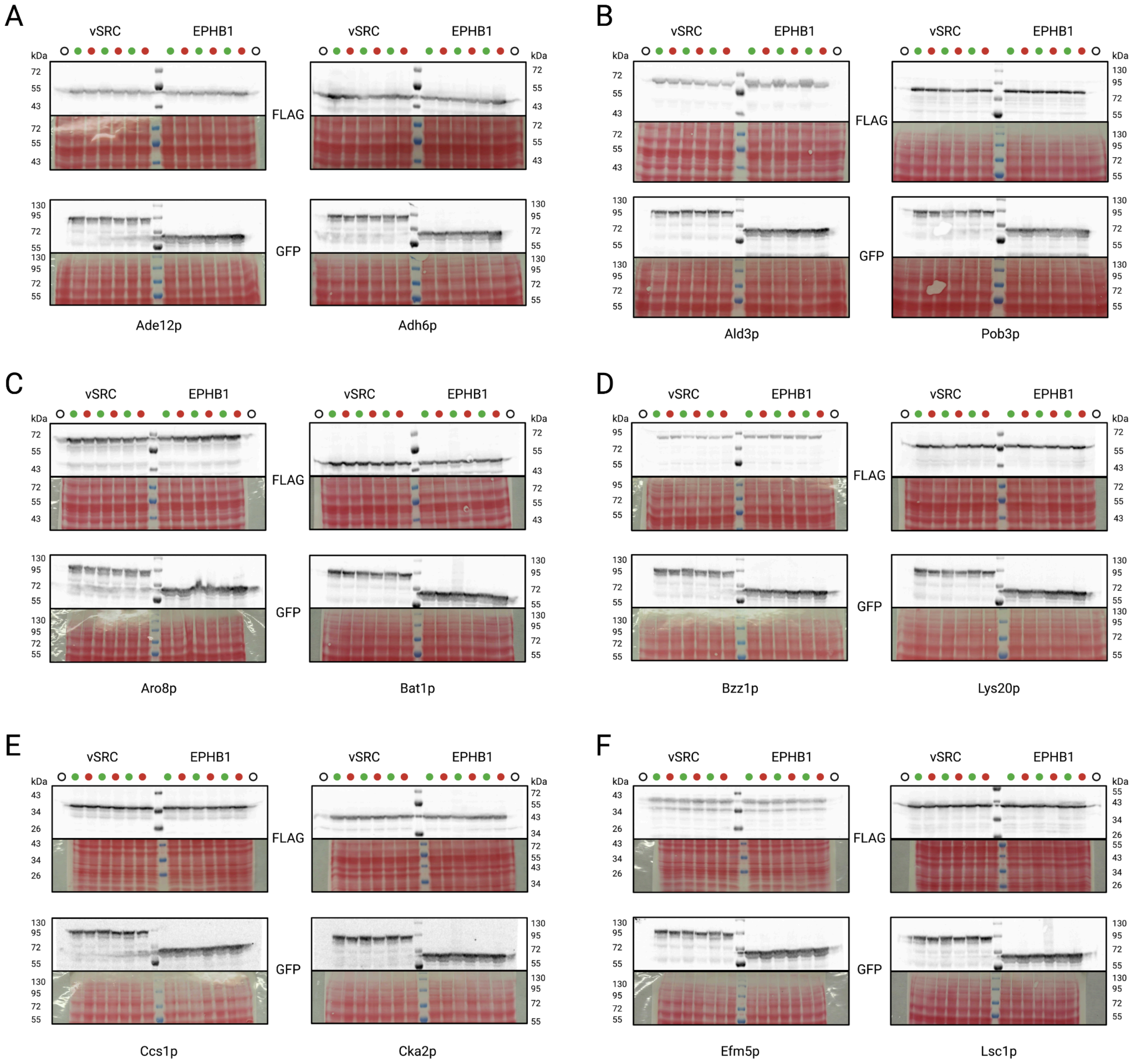

Appendix Figure S21: Protein with spurious phosphorylation sites analysed by Western Blot. For substrates with pY sites at intermediate-high stoichiometries and predicted to affect protein stability (high ΔΔG). Green dots show the active kinase version and red dots the kinase dead version. Empty circles show a negative control for the Western Blot. Protein **(A)** Ade12p, Adh6p, **(B)** Ald3p, Pob3p, **(C)** Aro8p, Bat1p, **(D)** Bzz1p, Lys20p, **(E)** Ccs1p, Cka2p, **(F)** Efm5p and Lsc1p were analysed. Cell pellets were prepared from three independent cultures. Proteins of interest were detected using an anti-Flag antibody. Kinases were detected using an anti-GFP antibody. Membranes were stained with Ponceau red to ensure proper protein transfer. Figure was created with Biorender.com.

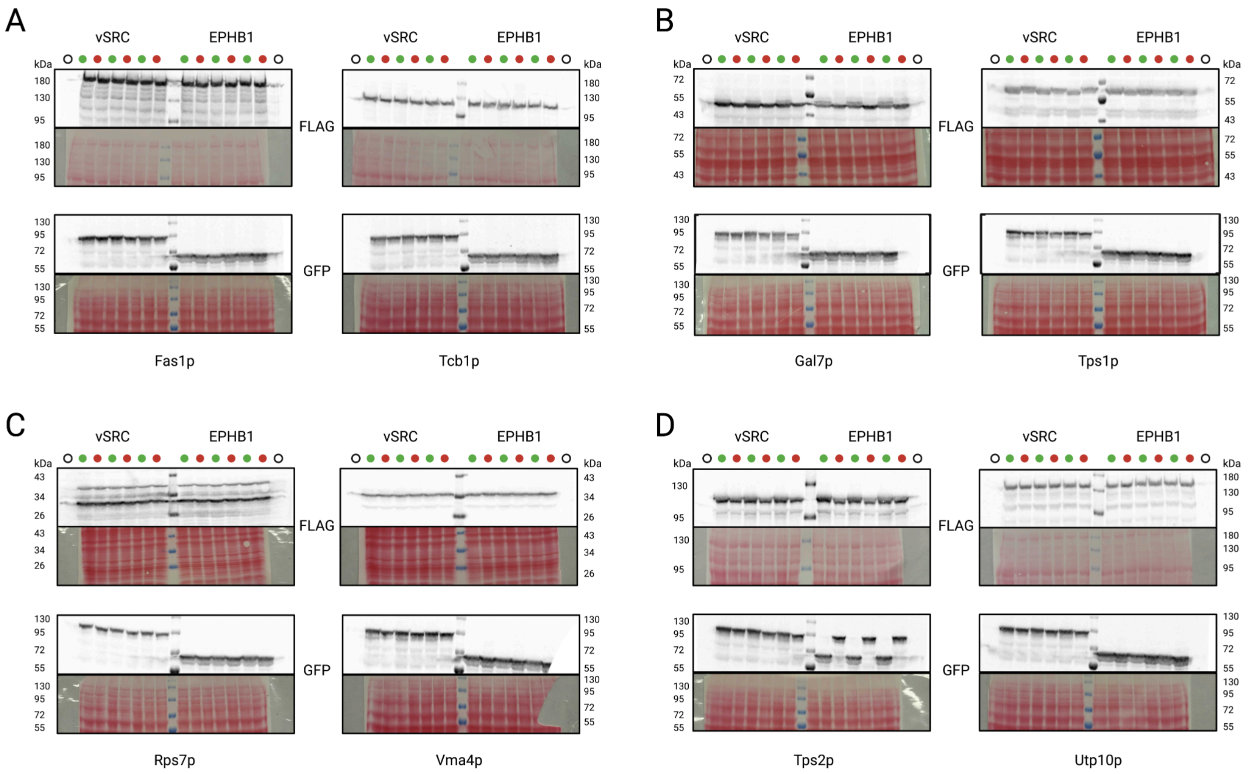

Appendix Figure S22: Protein with spurious phosphorylation site analysed by Western Blot. For substrates with pY sites at intermediate-high stoichiometries and predicted to affect protein stability (high ΔΔG). Green dots show the active kinase version and red dots the kinase dead version. Empty circles show a negative control for the Western Blot. Protein **(A)** Fas1p, Tcb1p, **(B)** Gal7p, Tps1p, **(C)** Rps7p, Vma4p, **(D)** Tps2p, and Utp10p were analysed. Cell pellets were prepared from three independent cultures. Proteins of interest were detected using an anti-Flag antibody. Kinases were detected using an anti-GFP antibody. Membranes were stained with Ponceau red to ensure proper protein transfer. Figure was created with Biorender.com.

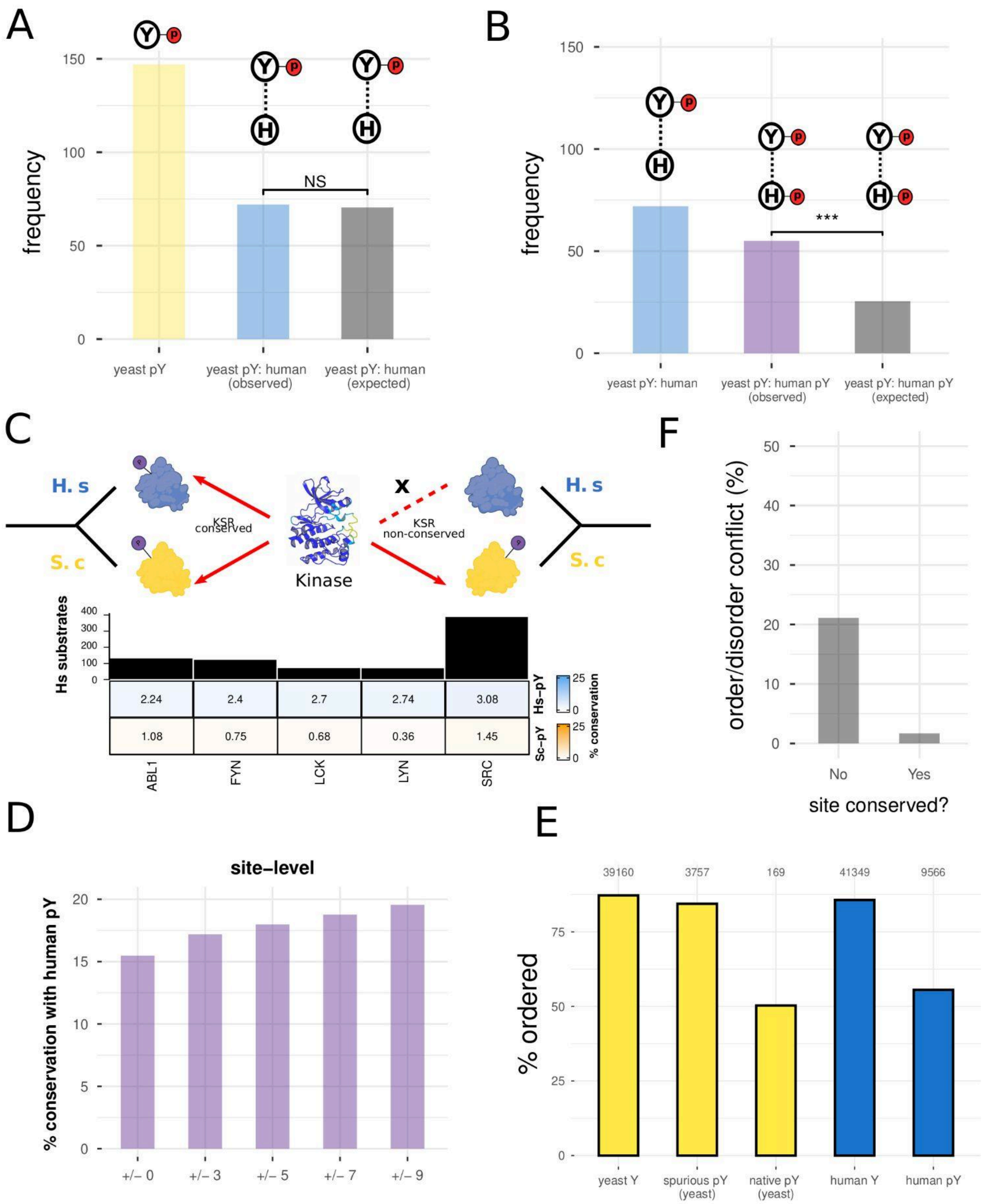

Appendix Figure S23: Supplementary analysis of conservation between spurious pY in yeast and native pY in humans. A) Human-yeast conservation at the whole protein level for native pY proteins in yeast. Yeast pY: number of unique native pY proteins in yeast. Yeast pY: human (observed): number of *observed* native pY proteins in yeast with at least one ortholog in human. Yeast pY: human (expected): number of *expected* unique native pY proteins in yeast with at least one ortholog in human, based upon the proportion of non-pY proteins that have at least one human ortholog. H symbol: human. Y symbol: yeast. P symbol: phosphosite. Dotted lines represent orthology relationships. **B)** Human-yeast conservation at the level of whole protein pY phosphorylation for native pY proteins in yeast. Yeast pY: human: number of *observed*

native pY proteins in yeast with at least one ortholog in human. Yeast pY: human pY (observed): number of *observed* native pY proteins in yeast with at least one ortholog in human that is Y-phosphorylated. Yeast pY: human pY (expected): number of *expected* native pY proteins in yeast with at least one ortholog in human that is Y-phosphorylated, based on non-pY proteins with a human ortholog and the proportion of those that are pY-phosphorylated. H symbol: human. Y symbol: yeast. P symbol: phosphosite. Dotted lines represent orthology relationships. **C)** conservation of kinase-substrate relationships (KSRs) between human and yeast. Top row (blue) is the proportion conserved relative to the sample size of known KSRs in humans. Bottom row (yellow) is the proportion conserved relative to the sample size of KSRs found in this study for yeast. **D)** Relating to the site-based conservation analysis in **Figure 5D,** changes in the % pY conservation (for yeast spurious pY substrate with at least one pY-phosphorylated ortholog in human) with increasing sizes of an alignment window centred around the spurious pY position in yeast. **E)** For yeast non-pY Y residues, spurious pY residues, native pY residues, human non-pY Y residues, and human pY residues, the percentage of sites predicted to be ordered in each group. **F)** For all pairs of human pY and yeast spurious pY on orthologous proteins, the percentage predicted as discordant in terms of the order/disorder prediction (order/disorder conflict).

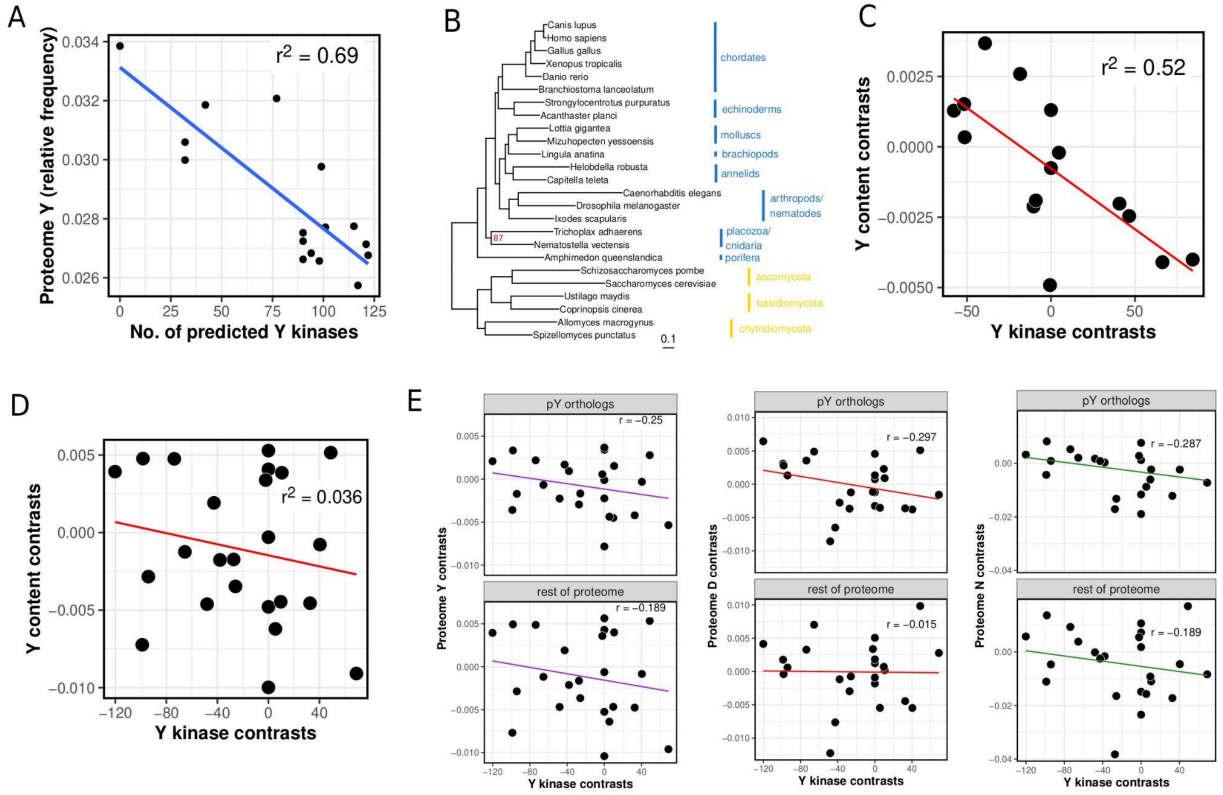

Appendix Figure S24: Supplementary analysis for testing metazoan counter-selection against pY at the level of the proteome. A) A reproduction of the (Tan *et al*, 2009) negative correlation between predicted Y kinases and proteomic Y content using modern proteome sequences and software for tyrosine kinase prediction. **B)** A species tree for all species used in the **Figure 6A-D** analysis (see Methods). The percentage of supporting ultrafast bootstrap replicates (/1000) was maximal (100%) except for the branch separating *N. vectensis* and *T. adhaerans* (87%). **C)** The result in panel **A** after applying a phylogenetic correction with the corresponding species tree using phylogenetic independent contrasts (Felsenstein, 1985). The species represented are the same as in (Tan *et al*, 2009). **D)** The result in **Figure 6A** after applying a phylogenetic correction with the corresponding species tree using phylogenetic independent contrasts (Felsenstein, 1985). The species used for this analysis are represented in panel **B. E)** Phylogenetically corrected correlation between the number of predicted tyrosine kinases (x-axis) and the proteome Y frequency (y-axis, left panel), D frequency (y-axis, middle panel), and N frequency (y-axis, right panel). In each case, the top panel represents the amino acid content only for orthologs of spurious pY substrates whereas the bottom panel represents the rest of the proteome (i.e. non-orthologs). The species used for this analysis are represented in panel **B.**

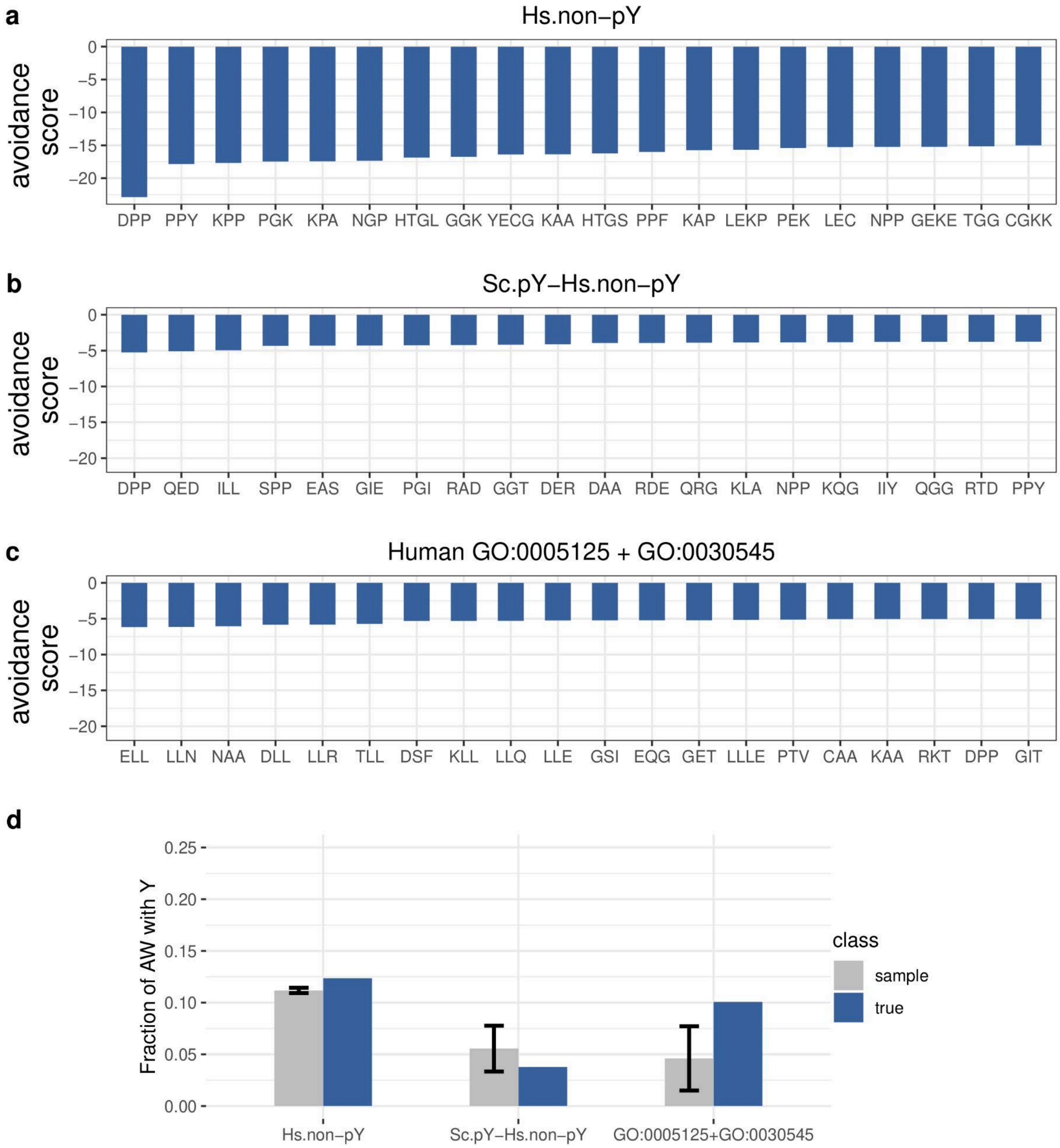

Appendix Figure S25: Analysis of 'avoided words' among protein sets of interest. A) Top 20 avoided words for human proteins not phosphorylated on tyrosine (Y). **B)** Top 20 avoided words for orthologs of spurious pY substrates (detected here) in *S. cerevisiae* that are not phosphorylated in humans. **C)** Top 20 avoided words for the gene ontology (GO) terms GO: 0005125 (cytokine activity) and GO: 0030545 (signalling receptor regulator activity) in humans that are enriched in tyrosine deserts. **D)** For each of the three protein sets described above, comparison of the number of avoided amino acid words containing tyrosine (Y) for the real data compared with a random sample from the human proteome generated 500 times with matched sample size. Grey bars and error bars represent the median and standard deviation, respectively.
