# Supplementary material for: The fitness cost of spurious phosphorylation
Source: EMBO J. 2024 Sep 10;43(20):4720–51. doi: 10.1038/s44318-024-00200-7 (PMC11480408; doi:10.1038/s44318-024-00200-7)
Supplement: Supplementary file 25 — Expanded View Figures [file 44318_2024_200_MOESM25_ESM.pdf]

Expanded View Figures

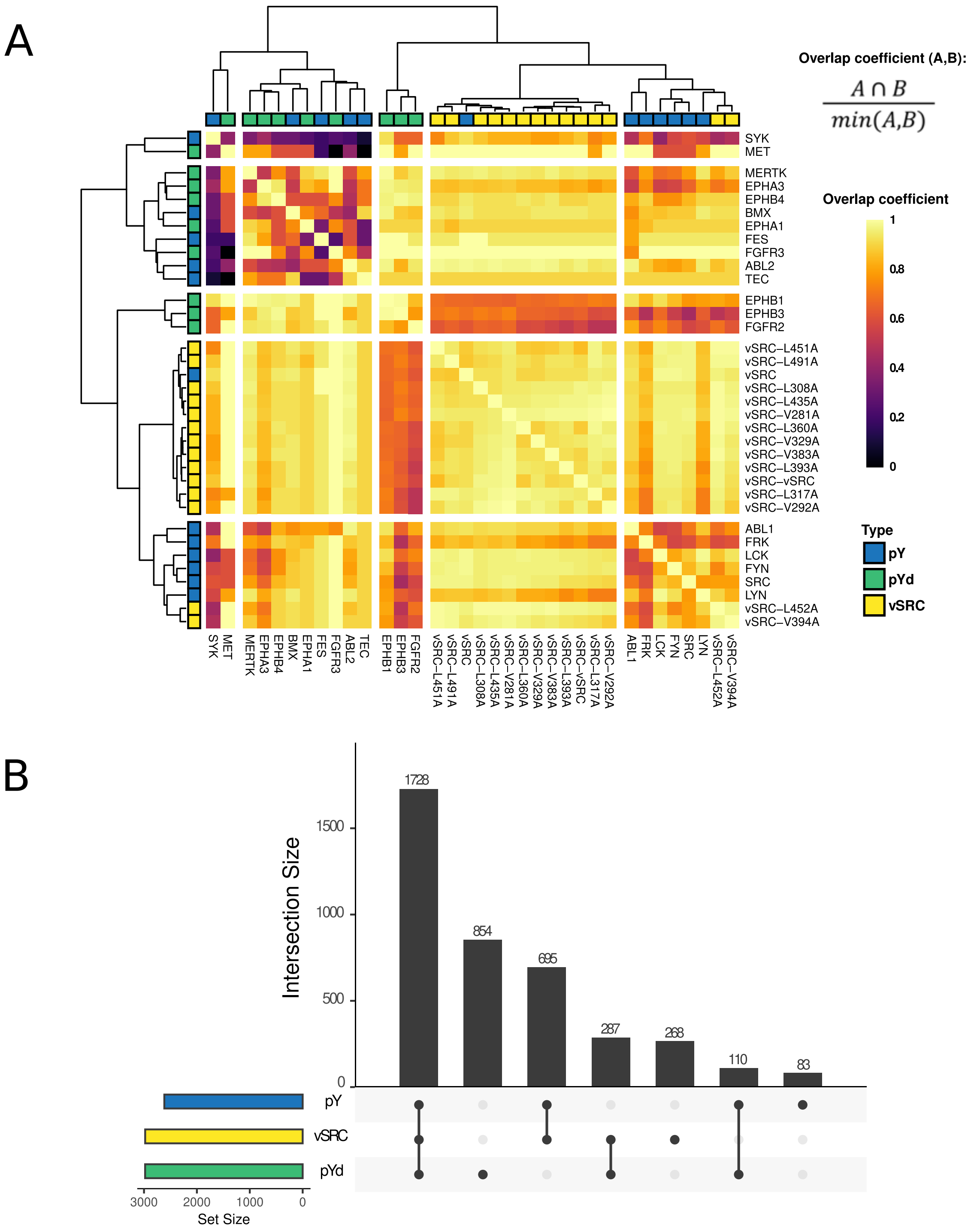

Figure EV1. Substrate overlap between different kinases expressed in yeast.

(**A**) Phosphoproteome overlap between the different kinase strains. Overlap is with respect to the unique upregulated (WT-dead) pY sites per kinase. Overlap is calculated in terms of the 'overlap coefficient' (top-right), which is the size of the intersection divided by the size of the smallest set. (**B**) Overlap between the pY sets above for the major pY, v-SRC, and pYd groups. Visualisation is in the form of an UpSet plot.

© The Author(s)

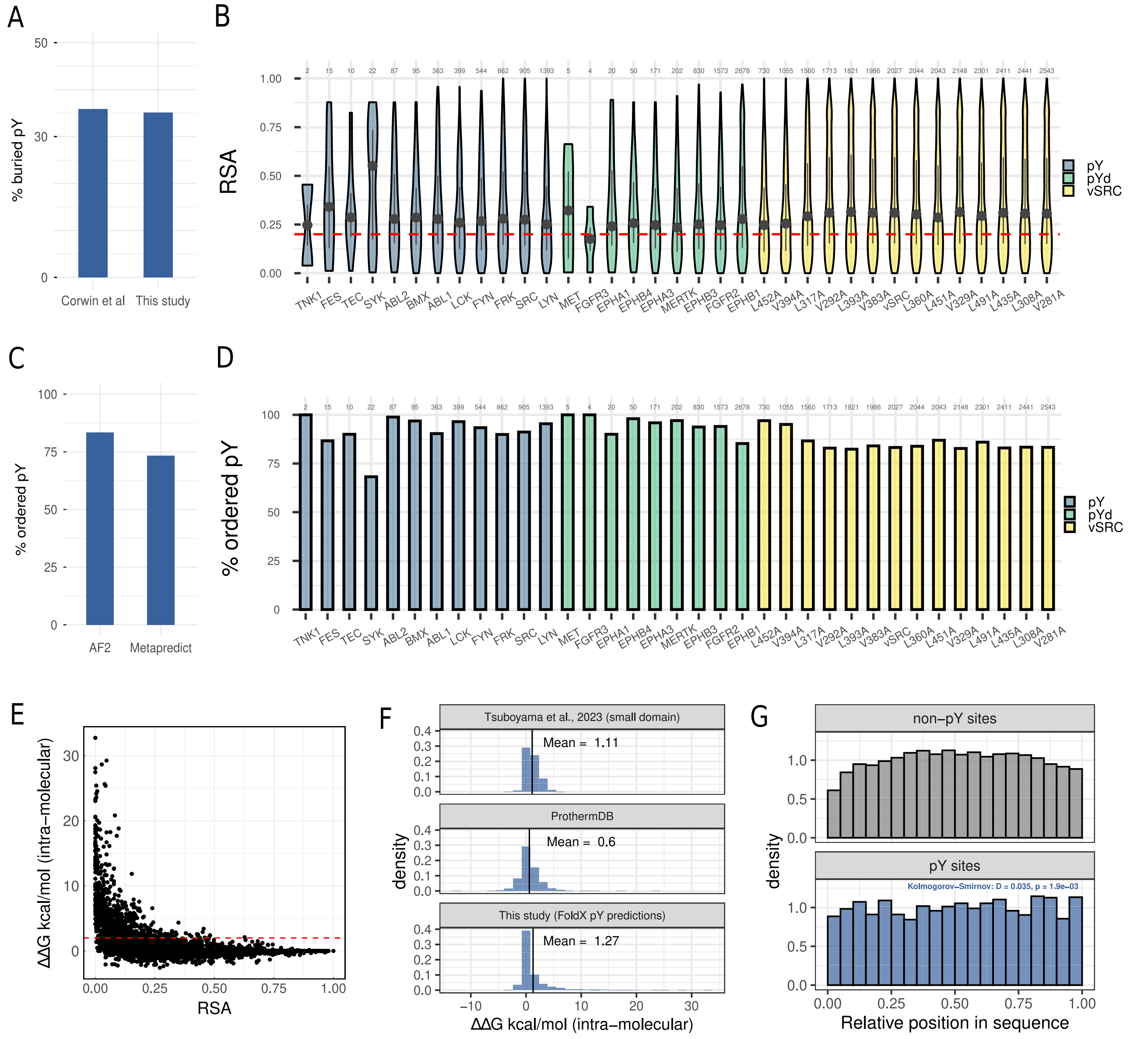

Figure EV2. Structural features of spurious phosphorylation.

(**A**) For all unique spurious pY reported in (Corwin et al, 2017) and this study, the percentage of sites predicted to be buried from AF2 structures based upon a relative solvent accessibility (RSA) threshold of 0.2 (**B**) The RSA of upregulated pY sites (WT-dead) for each kinase, divided by group. pY: full-length tyrosine kinases, pYd: tyrosine kinase domains, v-SRC: WT v-SRC and v-SRC mutants. The red dashed line corresponds to the cut-off for buried residues, set at an RSA of 0.2. The black point indicates the median (50th percentile) and the vertical lines connect the lower and upper quartiles (25th percentile and 75th percentile). Numbers above plots represent the number of unique upregulated pY sites (WT-dead) per kinase that could be mapped to an AF2 structure. (**C**) For all unique spurious pY phosphosites reported in this study, the percentage mapping to predicted ordered regions based upon the AF2 structures (left) and the sequence-based predictor Metapredict (right) (Emenecker et al, 2021). (**D**) The percentage of spurious pY sites mapping to ordered regions for each kinase, divided by group. Disorder/order predictions made upon the basis of the AF2 models (Akdel et al, 2022; Piovesan et al, 2022). (**E**) For each unique spurious pY that could be mapped to an AlphaFold2 model ($n = 3981$), the relationship between the RSA and the predicted ΔΔG of phosphorylation (Y to pY). The red dashed line corresponds to a ΔΔG threshold of 2 kcal/mol for destabilising pY. (**F**) ΔΔG (kcal/mol) distribution for the amino acid mutations reported in the empirical protease-based screen of (Tsuboyama et al, 2023) (top), a compilation of experimental ΔΔGs reported in ProThermDB for amino acid mutations (Nikam et al, 2021) (middle), and the ΔΔGs predicted in this study for spurious tyrosine phosphorylation. (**G**) Distribution of the relative pY position along the protein length for all unique spurious pY sites (pY) and non-phosphorylated sites on the same proteins (non-pY).

 © The Author(s)

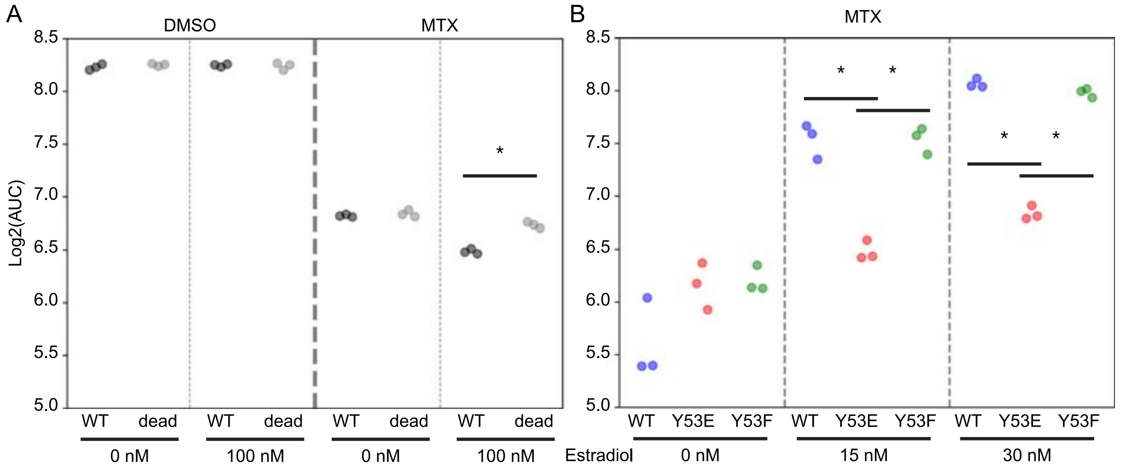

Figure EV3. Effect of EPHB1 expression on a specific PPI as measured by DHFR PCA.

(**A**) EPHB1 WT or dead was expressed in a strain to measure the interaction between Rvs167-DHFR[1,2| and Sla1-DHFR[3]. DHFR PCA is a method in which two interaction partners are fused to fragments of methotrexate (MTX) resistant DHFR. If both partners interact, the two fragments assemble into a functional DHFR and allow the growth of the yeast strain in the presence of MTX. The growth of the yeast strain is proportional to the amount of protein complex formed by the two partners. EPHB1 WT expression induces phosphorylation at position Y476 in Rvs167, which is predicted to destabilise the Rvs167-Sla1 interface at a ΔΔG of +2.15 kcal/mol, but without any predicted destabilisation of the Rvs167 fold (ΔΔG of −0.24 kcal/mol). Strains were grown in PCA media with or without MTX and with or without 100 nM estradiol to induce kinase expression. Strains were grown in triplicates for 72 h and the area under the curve (AUC) was calculated for the three replicates. A statistically significant difference (*t*-test, WT vs dead, $p = 3.45 \times 10^{-4}$, *) is shown on the graph. (**B**) $Rvs167_{SH3}$ with specific phosphomimetic mutations in the SH3 domain (Y53E and Y53F, corresponding to position Y476 in the full protein) was expressed with estradiol (15 or 30 nM). Y53F prevents phosphorylation; Y53E partially mimics the negative charge of the phosphate, but we caveat that the phosphate group on tyrosine has a larger negative charge and pY is very distinct structurally from the glutamate sidechain (Hunter, 2012; Reinhardt and Leonard, 2023). In this case, we only checked for the effect of the phosphomimetic mutants on the interaction between Rvs167 and Sla1, without the presence of the kinase. This removed all the fitness effects kinase expression could have on cell growth and focused only on the interaction destabilisation. Interaction with Sla1-DHFR[3] was tested for 72 h and AUC was calculated for the three replicates. Statistically significant differences (*t*-test, 15 nM estradiol *p* values WT vs Y53E $= 1.49 \times 10^{-3}$, 15 nM estradiol Y53F vs Y53E $= 5.89 \times 10^{-4}$, 30 nM estradiol WT vs Y53E $= 1.01 \times 10^{-5}$, 30 nM estradiol Y53F vs Y53E $= 1.25 \times 10^{-5}$, *) are shown on the graph. These results are in agreement with previous work demonstrating that tyrosine phosphorylation of this position in SH3 domains can perturb SH3 domain-dependent interactions (Dionne et al, 2018). However, we caution that the Y53E mutation is predicted to have a destabilising effect on the SH3 domain itself (ΔΔG of 2.03 kcal/mol), which would also contribute to the reduced formation of the Rvs167-Sla1 interaction (in addition to the destabilisation of the Rvs167-Sla1 interface). The figure was created with Biorender.com.

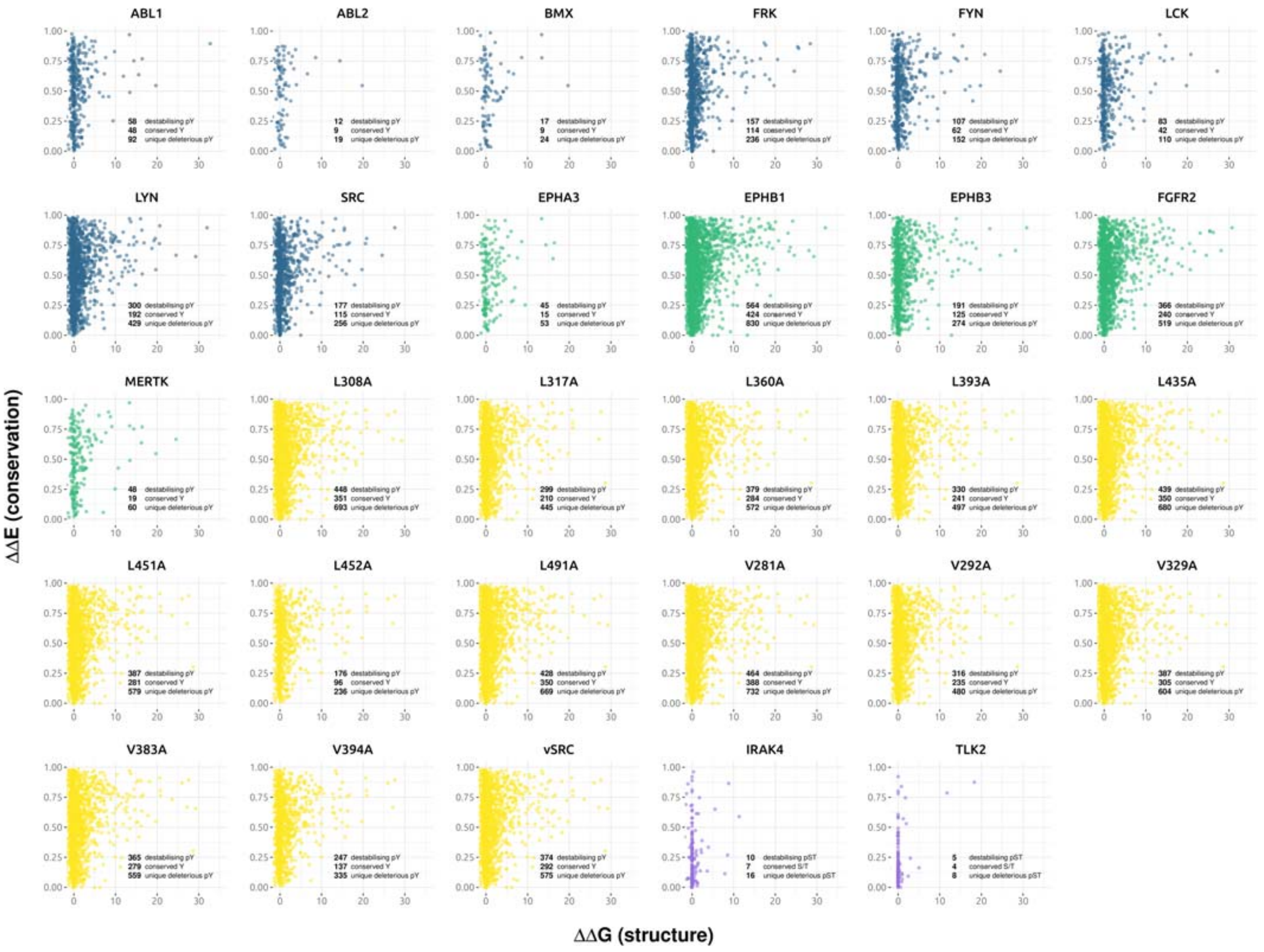

Figure EV4. Variant effect prediction (VEP) of spurious phosphorylation with respect to protein stability (x-axis) and sequence conservation (y-axis).

Higher ΔΔG values correspond to more destabilising pY (protein-level) whereas higher ΔΔE values correspond to pY mapping to more conserved Y positions. ΔΔG > 2 and ΔΔE > 0.8 were the thresholds used to determine deleterious pY via structure and conservation, respectively. Kinases are coloured by their groups: blue (full-length tyrosine kinases), green (tyrosine kinase domains), yellow (v-SRC and its mutants), and purple (pS/T kinases).

© The Author(s)

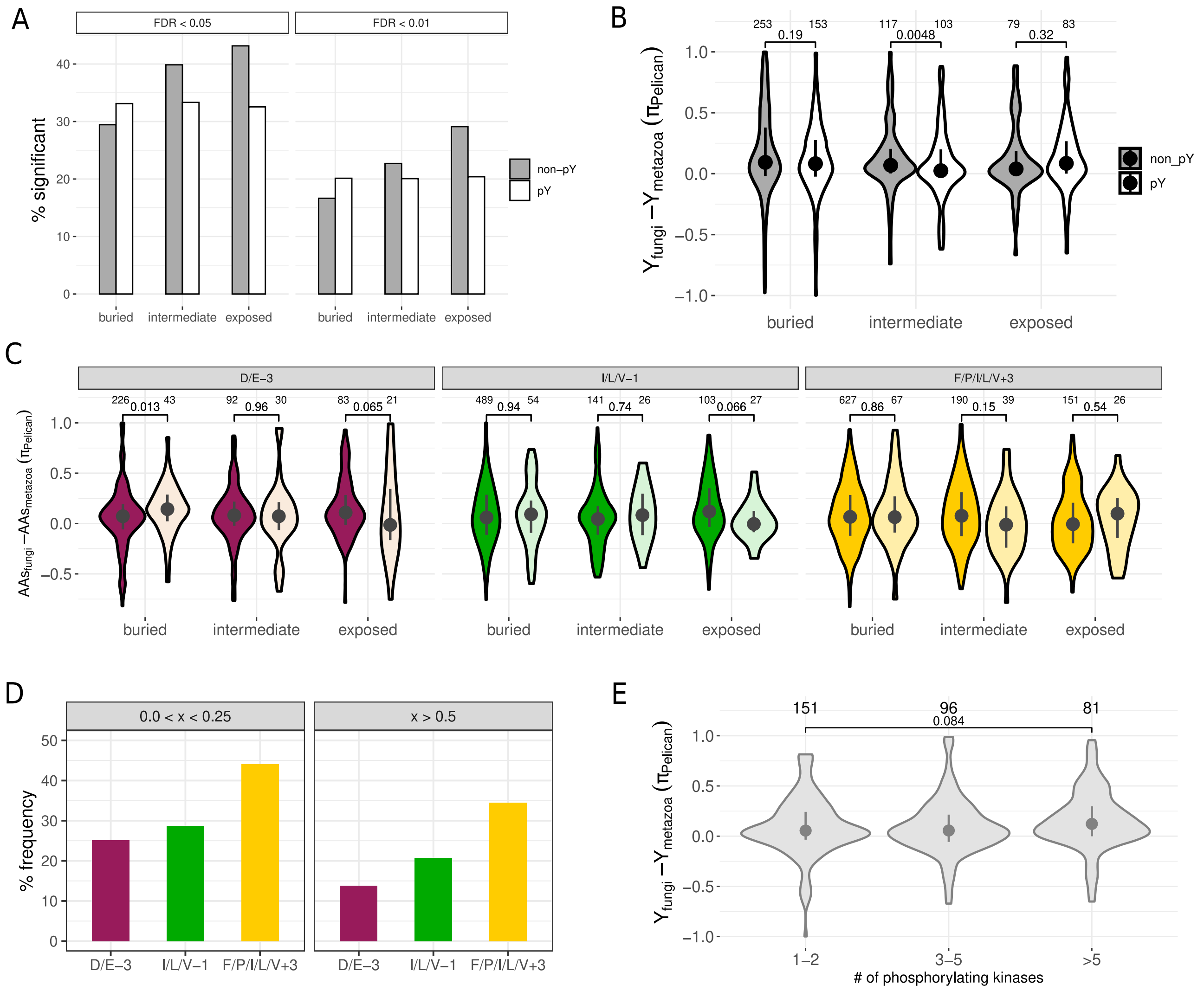
A
FDR < 0.05
FDR < 0.01
% significant
buried
intermediate
exposed
non−pY
pY
B
$Y_{fungi} - Y_{metazoa}$ ($\pi_{Pelican}$)
253 153 0.19
117 103 0.0048
79 83 0.32
buried
intermediate
exposed
non_pY
pY
C
D/E−3
I/L/V−1
F/P/I/L/V+3
$AAs_{fungi} - AAs_{metazoa}$ ($\pi_{Pelican}$)
226 0.013 43
92 0.96 30
83 0.065 21
489 0.94 54
141 0.74 26
103 0.066 27
627 0.86 67
190 0.15 39
151 0.54 26
buried
intermediate
exposed
D
0.0 < x < 0.25
x > 0.5
% frequency
D/E−3
I/L/V−1
F/P/I/L/V+3
E
151 96 81
0.084
$Y_{fungi} - Y_{metazoa}$ ($\pi_{Pelican}$)
1−2
3−5
>5
# of phosphorylating kinases

Figure EV5. Supplementary results for the site-based analysis of pY counter-selection in metazoan species.

(**A**) For spuriously phosphorylated tyrosines (pY) and non-phosphorylated tyrosines (non-pY), the percentage with a significant shift in amino acid profile between animal and fungal species, as inferred by Pelican software (Duchemin et al, 2023). (**B**) The same analysis as in Fig. 6F but after excluding non-pY sites that are poor candidates for Y phosphorylation on the basis of their sequence motif. Scores with a normalised motif score (0-1) maximum (across Y kinases) lower than 0.7 were excluded. Y kinase specificity models were constructed from data presented in (Sugiyama et al, 2019). The y-axis represents the difference between the inferred preference for Y in fungal species and metazoa species, calculated as their difference in equilibrium frequencies (π) by the Pelican software (Duchemin et al, 2023). The results are given for pY and non-pY tyrosines and separated according to their solvent accessibility (buried: RSA >0.2, intermediate: 0.2 < RSA <0.4, exposed: RSA >0.4). Sample sizes are buried non-pY ($n = 253$), buried pY ($n = 153$), intermediated non-pY ($n = 117$), intermediate pY ($n = 103$), exposed non-pY ($n = 79$), exposed pY ($n = 83$). The black point indicates the median (50th percentile) and the vertical lines connect the lower and upper quartiles (25th percentile and 75th percentile). In each case, a two-sided Kolmogorov–Smirnov test was performed. (**C**) Testing for counter-selection against residues that are often found in Y kinase phosphorylation motifs: D/E-3, I/L/V-1, and F/P/I/L/V + 3 (Deng et al, 2014; Li et al, 2023; Sugiyama et al, 2019). The analysis was performed and presented the same way as it is described in Fig. 6F for the central Y residue. The y-axis represents the difference between the inferred preference for the motif amino acids (D + E at position -3, or I + L + V at position-1, or F + P + I + L + V at position +3) in fungal and metazoan species, calculated as their difference in equilibrium frequencies (π) by the Pelican software (Duchemin et al, 2023). The black point indicates the median (50th percentile) and the vertical lines connect the lower and upper quartiles (25th percentile and 75th percentile). The results are separated according to their solvent accessibility (buried: RSA > 0.2, intermediate: 0.2 < RSA <0.4, exposed: RSA >0.4). The non-phosphorylated sites are in the darker colour for all three panels. The sample sizes above the plots indicate the number of unique sites that fall into each category in terms of their phosphorylation status (non-pY vs pY), motif (D/E-3, I/L/V-1, and F/P/I/L/V + 3), and accessibility (buried, intermediate, exposed). In each case, a two-sided Kolmogorov–Smirnov test was performed to determine statistical significance. (**D**) Determining whether these motif residues are more likely to be found in strongly counter-selected Ys (right) compared to weakly counter-selected Ys (left). The 'x' parameters refer to the difference in inferred Y preference between fungal and animal species (π_{fungi} - $\pi_{metazoa}$), with values closer to 1 being stronger candidates for Y counter-selection in animal species. (**E**) Test to determine if sites that are phosphorylated by many Y kinases in this dataset are more strongly counter-selected than sites that are phosphorylated by only a small number of kinases. The analysis was performed and presented the same way as it is described in Fig. 6F. The y-axis represents the difference between the inferred preference for Y in fungal species and metazoa species, calculated as their difference in equilibrium frequencies (π) by the Pelican software (Duchemin et al, 2023). The black point indicates the median (50th percentile) and the vertical lines connect the lower and upper quartiles (25th percentile and 75th percentile). The x-axis represents the number of unique kinases targeting the Y phosphosite. Sample sizes indicate the number of upregulated pY (WT-dead) in each category. This analysis excludes the v-SRC mutants, which overlap strongly in terms of their substrate profiles. The *p* value was inferred from a one-sided Kolmogorov–Smirnov test.

© The Author(s)
